# Isolation of a natural DNA virus of *Drosophila melanogaster*, and characterisation of host resistance and immune responses

**DOI:** 10.1371/journal.ppat.1007050

**Published:** 2018-06-04

**Authors:** William H. Palmer, Nathan C. Medd, Philippa M. Beard, Darren J. Obbard

**Affiliations:** 1 Institute of Evolutionary Biology University of Edinburgh, Charlotte Auerbach Road, Edinburgh, United Kingdom; 2 The Roslin Institute and The Royal (Dick) School of Veterinary Studies, University of Edinburgh, Midlothian, United Kingdom; 3 Centre for Infection, Evolution and Immunity, University of Edinburgh, Edinburgh, United Kingdom; CNRS-UPR9022 France, FRANCE

## Abstract

*Drosophila melanogaster* has played a key role in our understanding of invertebrate immunity. However, both functional and evolutionary studies of host-virus interaction in *Drosophila* have been limited by a dearth of native virus isolates. In particular, despite a long history of virus research, DNA viruses of *D*. *melanogaster* have only recently been described, and none have been available for experimental study. Here we report the isolation and comprehensive characterisation of Kallithea virus, a large double-stranded DNA virus, and the first DNA virus to have been reported from wild populations of *D*. *melanogaster*. We find that Kallithea virus infection is costly for adult flies, reaching high titres in both sexes and disproportionately reducing survival in males, and movement and late fecundity in females. Using the *Drosophila* Genetic Reference Panel, we quantify host genetic variance for virus-induced mortality and viral titre and identify candidate host genes that may underlie this variation, including *Cdc42-interacting protein 4*. Using full transcriptome sequencing of infected males and females, we examine the transcriptional response of flies to Kallithea virus infection and describe differential regulation of virus-responsive genes. This work establishes Kallithea virus as a new tractable model to study the natural interaction between *D*. *melanogaster* and DNA viruses, and we hope it will serve as a basis for future studies of immune responses to DNA viruses in insects.

## Author summary

The fruit fly *Drosophila melanogaster* is a useful model species to study host-virus interaction and innate immunity. However, few natural viruses of *Drosophila* have been available for experiments, and no natural DNA viruses of *Drosophila melanogaster* have been available at all. Although infecting flies with viruses from other insects has been useful to uncover general immune mechanisms, viruses that naturally infect wild flies could help us to learn more about the coevolutionary process, and more about the genes that underlie host-virus interactions. Here we present an isolate of a DNA virus (named Kallithea virus) that naturally infects the model species *Drosophila melanogaster* in the wild. We describe the basic biology of infection by this virus, finding that both male and female flies die from infection. We also find that females are more tolerant of infection than males, but lay fewer eggs than uninfected females. We quantify genetic variation for virus resistance in the flies, and we use RNA sequencing to find which genes are expressed in male and female flies in response to infection. These results will form the basis for further research to understand how insects defend themselves against infection by DNA viruses, and how DNA viruses can overcome antiviral defence.

## Introduction

Studies of *Drosophila melanogaster* are central to our understanding of infection and immunity in insects. Moreover, many components of the *Drosophila* immune response, including parts of the JAK-STAT, IMD, and Toll (and perhaps RNA interference; RNAi) pathways are conserved from flies to mammals [1–8], making *Drosophila* a valuable model beyond the insects. The experimental dissection of antiviral immune pathways in *Drosophila* has benefited from both natural infectious agents of *Drosophila*, such as Drosophila C Virus (DCV) and Sigma virus (DmelSV), and from artificial infections, such as Cricket paralysis virus (isolated from a cricket), Flock House Virus (from a beetle), Sindbis virus (from a mosquito) and Invertebrate Iridescent Virus 6 (from a moth). However, while the availability of experimentally tractable, but non-natural, model viruses has been a boon to studies of infection, it also has two potential disadvantages. First, the coevolutionary process means that pairs of hosts and pathogens that share a history may interact very differently to naive pairs (e.g. [9,10]). For example, the Nora virus of *D*. *immigrans* expresses a viral suppressor of RNAi that is functional in the natural host, but not in *D*. *melanogaster* [11]. Second, if our aim is to understand the coevolutionary process itself, then the standing diversity in both host and virus populations may be fundamentally altered in coevolving as opposed to naïve pairs. For example, heritable variation for host resistance was detectable for two natural viruses of *D*. *melanogaster*, but not for two non-natural viruses [12,13]. This difference was in part due to large-effect segregating polymorphisms for resistance to the natural viruses, which are predicted to result from active coevolutionary dynamics [14–16].

Experimental studies of host-virus interaction using *Drosophila* have consequently been limited by a lack of diverse natural virus isolates. In particular, no natural DNA viral pathogens of *D*. *melanogaster* have previously been isolated ([17,18]; but see [19] for a DNA virus of *Drosophila innubila*), and all natural (and most artificial) studies of viral infection in *D*. *melanogaster* have therefore focussed on the biology of RNA viruses and resistance to them [20,21]. For DNA viruses, our molecular understanding of insect-virus interaction has instead been largely shaped by the response of lepidopterans to their natural baculoviruses. These are often of agronomic and/or ecological importance [22], but lack the genetic toolkit of *D*. *melanogaster*. Nevertheless, Lepidopteran studies of the expression response to baculovirus infection have implicated host genes with a diverse array of functions, including cuticle proteins, reverse transcriptases, and apoptotic factors, suggesting previously uncharacterised and/or host-specific antiviral immune mechanisms [23–26].

To date, the only DNA virus studies in *D*. *melanogaster* have used Insect Iridescent Virus 6 (IIV6), an enveloped dsDNA moth iridovirus with a broad host range [27]. This work has shown that *Drosophila* RNAi mutants are hyper-susceptible to IIV6 infection, and that IIV6 encodes a viral suppressor of RNAi, indicating that at least some immune responses to DNA viruses overlap with those to RNA viruses [21,28,29]. However, while IIV6 injections are lethal in *D*. *melanogaster*, and IIV6 has provided useful information about the *Drosophila* response to DNA viruses, for the reasons described above it is hard to interpret the implications of this for our understanding of natural host-virus interaction.

Metagenomic sequencing has recently identified several natural dsDNA nudivirus infections in wild-caught *Drosophila*, including in *D*. *innubila* (*D*. *innubila* Nudivirus, DiNV; [19,30]) and in *D*. *melanogaster* and *D*. *simulans* (‘Kallithea virus’, KV [31]; ‘Esparto virus’ (KY608910.1), and ‘Tomelloso virus’ (KY457233.1)), and also ssDNA densovirus infections in *D*. *melanogaster* and *D*. *simulans* (‘Vesanto virus’ (KX648534.1), ‘Linvill Road virus’ (KX648536.1), and ‘Viltain virus’ (KX648535.1)) [32]. Like other members of the *Nudiviridae*, DiNV and KV are enveloped dsDNA viruses of around 120-230Kbp with 100–150 genes. This recently-recognised family forms a clade that is either sister to, or paraphyletic with, the Bracoviruses [33] that have been ‘domesticated’ by Braconid parasitoid wasps following genomic integration, and now provide essential components of the wasp venom [34,35]. Together, the nudiviruses and bracoviruses are sister to the baculoviruses, which are arguably the best-studied dsDNA viruses of insects. They share many of their core genes with baculoviruses, but canonically lack occlusion bodies [36]. PCR surveys of wild flies suggest that DiNV is common in several species in the subgenus *Drosophila*, and that KV is widespread and common in *D*. *melanogaster* and *D*. *simulans*, being detectable in 10 of 17 tested populations, with an estimated global prevalence of 2–7% [31]. However, we currently know little about the interaction between these viruses and their hosts. Indeed, although studies of wild-caught *D*. *innubila* individuals infected by DiNV suggest that infection is costly [19], in the absence of an experimental *D*. *melanogaster* nudivirus isolate, it has not been possible to capitalise the power of *D*. *melanogaster* genetics to further elucidate the costs associated with infection, or the genetic basis of resistance.

Here we present the isolation of KV from wild-collected *D*. *melanogaster* via passage in laboratory stocks and gradient centrifugation. We use this isolate to characterise the fundamental phenotypic impacts of infection on host longevity and fecundity. We then use the *Drosophila* Genetic Reference Panel (DGRP; [37]) to quantify and dissect genetic variation in immunity to KV infection in males and females, and use RNA sequencing analyses of an inbred line to quantify host and virus transcriptional response in both sexes. We find that KV causes higher rates of mortality following injection in males, but that males have lower viral titre, suggesting some female tolerance to infection. However, we also find that female movement is decreased following infection, and that infected females have significantly reduced late-life fecundity—highlighting the importance of considering infection phenotypes beyond longevity. We find a genetic correlation in longevity between KV-infected males and females, and a weak negative genetic correlation between mortality and KV titre in females, and we report host loci that have variants significantly associated with each trait. Finally, our expression analysis of infected individuals supports a dramatic cessation of oogenesis following infection, and significant differential regulation of serine proteases and certain immune genes. This work establishes KV as a new natural model for DNA virus infection in *D*. *melanogaster* and will enable further dissection of the insect antiviral immune response.

## Materials and methods

### Isolation of Kallithea virus

We identified KV-infected flies through a PCR screen for previously published *D*. *melanogaster* viruses in 80 previously untested wild-caught flies (see [31] for primers and cycling conditions). We homogenised each fly in 0.1 mL of Ringer's solution, transferred half of the homogenate to Trizol for nucleic acid extraction, and performed RT PCR assays on the resulting RNA for all *D*. *melanogaster* viruses reported by Webster *et al* [31]. We selected a KV-positive sample from Thika, Kenya (Collected by John Pool in 2009; subsequently stored at -80C), removed debris from the remaining fly homogenate by centrifugation for 10 minutes at 1000 × g, and microinjected 50 nL of the supernatant into *Dicer-2*^*L811fsX*^ flies, which lack a robust antiviral immune response [38]. After one week, we homogenised 100 KV-injected *Dicer-2* ^*L811fsX*^ flies in 10 uL Ringer’s solution per fly, cleared the solution by centrifugation as above, and re-injected this homogenate into further *Dicer-2*^*L811fsX*^ flies. This process was then repeated twice more with the aim of increasing viral titres. In the final round of serial passage, we injected 2000 *Dicer-2* ^*L811fsX*^ flies, which were homogenised in 5 mL 10 mM Tris-HCl. We cleared the homogenate by centrifuging at 1000 × g for 10 minutes, filtering through cheese cloth, centrifuging twice more at 6000 × g for 10 minutes, and finally filtering through a Millex 0.45 µm polyvinylidene fluoride syringe filter. The resulting crude virus preparation was used as input for gradient ultracentrifugation.

We screened the crude preparation by RT-PCR for other published *Drosophila* virus sequences, and identified the presence of DAV, Nora virus, DCV, and La Jolla virus. To separate KV from these viruses, we used equilibrium buoyant density centrifugation in iodixanol (“OptiPrep”, Sigma-Aldrich) as enveloped viruses are expected to have lower buoyant densities than most unenveloped viruses. Iodixanol is biologically inert, and gradient fractions can be used directly for downstream infection experiments (avoiding dialysis, which we found greatly reduces KV titres). We concentrated virus particles by centrifuging crude virus solution through a 1 mL 10% iodixanol layer onto a 2 mL 30% iodixanol cushion at 230,000 × g for 4 hours in a Beckman SW40 rotor. Virus particles were taken from the 30%-10% interphase, and layered onto a 40%-10% iodixanol step gradient, with 2% step changes, and centrifuged for 48 hours at 160,000 × g. We fractionated the gradient at 0.5 mL intervals, phenol-chloroform extracted total nucleic acid from aliquots of each fraction, and measured virus concentration by quantitative PCR (qPCR). We pooled all Kallithea-positive, RNA virus-negative fractions and calculated the infectious dose 50 (ID50) by injecting 3 vials of 10 flies with each of a series of 10-fold dilutions and performing qPCR after 5 days. We simultaneously performed the above isolation protocol with uninfected *Dicer-2*^*L811fsX*^ flies and extracted the equivalent fractions for use as an injection control solution (hereafter referred to as “gradient control”).

### Transmission electron microscopy

A droplet of viral suspension was allowed to settle on a Formvar/Carbon 200 mesh Copper grid for 10 minutes. We removed excess solution and applied a drop of 1% aqueous uranyl acetate for 1 minute before removing the excess by touching the grid edge with filter paper. The grids were then air dried. Samples were viewed using a JEOL JEM-1400 Plus transmission electron microscope, and representative images were collected on a GATAN OneView camera.

### Measurement and analysis of viral titre

Flies were reared on a standard cornmeal diet until infection, after which they were transferred to a solid sucrose-agar medium. We infected flies by abdominal injection of 50 nL of 10^5^ ID50 KV using a Nanoject II (Drummond Scientific), and these flies were then used to assay changes in viral titre, mortality, fecundity, or daily movement. To test whether the change in viral titre over time was influenced by sex or the presence of *Wolbachia* endosymbionts, we injected 25 vials of 10 male or female *Oregon R* flies with KV, with or without *Wolbachia* (totalling 1000 flies). We phenol-chloroform extracted total nucleic acid at 5 time-points: directly after injection and 3, 5, 10, and 15 days post-infection. We used qPCR to measure viral titre relative to copies of the fly genome with the following (PCR primers: kallithea_126072F CATCAATATCGCGCCATGCC, kallithea_126177R GACCGAGTTAGCGTCAATGC, rpl32_465F CTAAGCTGTCGGTGAGTGCC, rpl32_571R: TGTTGTCGATACCCTTGGGC). We analysed the log-transformed relative expression levels of Kallithea virus as a Gaussian response variable in a linear mixed model using the Bayesian generalised mixed modelling R package MCMCglmm (V2.24; [39]). R code and raw data used to fit all models in this paper is provided on figshare (doi: 10.6084/m9.figshare.c.3936037.v1).

The fixed effects portion of the model included an intercept term and coefficients for the number of days post-inoculation (DPI), sex, and DPI by sex interaction. We estimated random effects for each qPCR plate and assumed random effects and residuals were normally distributed. We initially fitted the model with *Wolbachia* infection status included as a fixed effect, however this term was not significant and was excluded from the final model.

We also attempted to infect flies with KV by feeding. We anesthetised flies in an agar vial and sprayed 50 uL of 5x10^3^ ID50 KV onto the flies and food. We then collected flies immediately (for the zero time-point) and at 7 DPI and used the primers above to calculate relative KV titre.

### Mortality following KV infection

We performed mortality assays to test the effect of KV infection on longevity, and to test whether this was affected by sex or *Wolbachia* infection status. We injected a total of 1200 *Oregon R* flies with control gradient or KV for each sex with or without *Wolbachia* (*Wolbachia* had previously been cleared by 3 generations of Ampicillin treatment and its absence was confirmed by PCR). We maintained flies for each treatment in 10 vials of 10 flies, and recorded mortality daily for three weeks. Mortality that occurred in the first day after infection was assumed to be due to the injection procedure and excluded from further analysis. We analysed mortality using an event-analysis framework as a generalised linear mixed model using MCMCglmm, with per-day mortality in each vial as a binomial response variable. We included fixed effects for DPI, DPI^2^ (used to capture nonlinear mortality curves), KV infection status, the two-way interaction between DPI and KV infection status, the two-way interaction between DPI and sex, and the three-way interaction between DPI, KV infection status, and sex. We fitted vial as a random effect to account for non-independence among flies within vials, assuming these follow a normal distribution. As in the model for viral titre, we found no evidence for differences associated with *Wolbachia* infection, and *Wolbachia* terms were excluded from the final model. The higher rate of male mortality we observed was also confirmed in a second independent experiment using an outbred population derived from the Drosophila Genetic Reference Panel (DGRP; see below).

### Fecundity following KV infection

We measured fecundity during early (1 and 2 DPI) and late (7 and 8 DPI) Kallithea virus infection. Virgin female flies from an outbred population derived from the DGRP ([37]; created from 113 DGRP lines and maintained at a low larval density with non-overlapping generations) were injected with either KV, or with chloroform-inactivated KV as a control, and individually transferred to standard cornmeal vials. The following day we introduced a single male fly into the vial with the virgin female. We transferred the pair to new vials each day and recorded the number of eggs laid. Per-day fecundity was analysed in MCMCglmm as a Poisson response variable using a hurdle model, which models the probability of zeroes in the data and the Poisson process as separate variables. We included fixed effects associated with KV infection status, infection stage (early or late), the interaction between KV infection and infection stage, and random effects associated with each fly pair (vial).

We analysed ovary morphology to examine whether changes in fecundity were detectable in ovaries. Flies were injected with either control virus solution or KV and kept on solid sucrose-agar medium vials. After 8 DPI, flies were transferred to vials with standard cornmeal medium supplemented with yeast. Two days later, we dissected ovaries in phosphate-buffered saline solution, fixed ovaries in 4% paraformaldehyde, and stained nuclei with DAPI. Ovaries were analysed under a Leica fluorescence microscope, and we recorded whether each ovariole within an ovary included egg chambers past stage 8 (i.e. had begun vitellogenesis), and whether any egg chambers within an ovariole exhibited apoptotic nurse cells. The probability of an ovariole containing a post-vitellogenic egg chamber was analysed using a logistic regression in MCMCglmm, with KV infection status as a fixed effect and the ovary from which the ovariole derived as a random effect. We analysed whether apoptotic nurse cells are associated with KV virus-infected ovary in the same way.

### Daily movement following KV infection

We used a *Drosophila* Activity Monitor (DAM, TriKinetics; [40]) to measure per-day total movement of individual flies. The DAM is composed of multiple hubs, each with 32 tubes containing a single fly, and movement is recorded on each occasion the fly breaks a light beam. We injected 96 female flies from an outbred DGRP population with either chloroform-inactivated KV or KV, randomly assigned these flies within and across 3 hubs, and measured total movement for one week. Movement was binned for each day and this per-day total movement was analysed in a linear mixed model as a Poisson response variable using MCMCglmm. We completely excluded flies that failed to move for a whole day or longer, assuming them to be dead. As before, we included fixed effects associated with DPI, KV infection status, and the interaction between KV and DPI. We included random effects associated with each fly (repeated measures) and each of the DAM hubs, and assumed each of these take values from a normal distribution.

### Quantitative genetic analysis

The DGRP is a collection of highly inbred fly lines derived from a *D*. *melanogaster* population collected in Raleigh, North Carolina [37], and is widely used to estimate and dissect genetic variation in complex traits in *Drosophila*. We measured KV titre in females and mortality following KV infection in both sexes for 125 DGRP lines, and estimated genetic (line) variances and covariances among these traits. To measure viral titre in the DGRP, we infected 5 vials of 10 flies for each line across 5 days, with a vial from each line being represented each day. After 8 DPI, living flies were killed and homogenised in Trizol for nucleic acid extraction and qPCR. To measure mortality following KV infection in the DGRP, we injected 3 vials of 10 flies of each sex and recorded mortality on alternate days until half the flies in the vial were dead (i.e. median survival time). Flies were transferred to fresh agar vials every 10 days. Mortality occurring in the first 3 DPI was assumed to be caused by the injection procedure and was removed from the analysis.

We fitted a multi-response linear mixed model in MCMCglmm to estimate heritability and genetic covariances among lines

$$y_{iklpqr}^{trait}=\beta_{p}^{trait}+\beta_{pk}^{trait:sex}+\mu_{pq}^{trait:date}+\mu_{l}^{plate}+\mu_{pkr}^{trait:sex:line}+\varepsilon_{iklpqr}^{trait:sex}$$ [1]

where $y_{iklpqr}^{trait}$ is the log-transformed relative viral titre or the duration until median mortality (LT50). We only estimated sex-specific fixed effects ($\beta^{trait:sex}$) for LT50, because we did not measure titre in both sexes. The first part of the random effects model accounts for block effects due to date of injection ($\mu^{trait:date}$) and qPCR plate ($\mu^{plate}$). We assumed a 2x2 identity matrix as the covariance structure for $\mu^{trait:date}$, with effects associated with each trait from independent normal distributions. Effects for the $l^{th}$ plate were assumed to be normally distributed. The second part of the random effects model ($\mu^{trait:sex:line}$) estimates the variance in each trait across lines and was allowed to vary by sex. We estimated all variance-covariance components of the 3x3 G matrix associated with $\mu^{trait:sex:line}$. Finally, we fitted separate error variances for each trait in each sex ($\varepsilon^{trait:sex})$, where residuals were associated with independent normal distributions.

The diagonal elements of the $\mu^{trait:sex:line}$ covariance matrix represent posterior distributions of genetic variances for viral titre in females, LT50 in females, and LT50 in males ($V_{G}^{titre}$, $V_{G}^{mortality♀},$ $V_{G}^{mortality♂}$). We calculated broad-sense heritability (i.e. line effects) for each trait as $H^{2}=\frac{V_{G}}{V_{G}+V_{R}}$, where $V_{R}$ is the residual variance associated with each trait, estimated in the model as $\varepsilon^{trait:sex}$. However, heritabilities cannot readily be compared because of their dependence on the residual variance, which can be vastly different for different phenotypes [41]. Therefore, we also calculated the coefficient of genetic variation (CV_G_) as ${CV}_{G}=\frac{100*\sqrt{V_{G}}}{\mu }$, where V_G_ is standardised by the phenotypic mean (μ) and is more appropriate for comparisons across phenotypes. All confidence intervals reported are 95% highest posterior density intervals.

### Genome-wide association studies

We used measurements of viral titre and mortality following KV infection in the DGRP lines to perform a series of genome-wide association studies (GWAS). Although our power to detect small-effect genetic variants with only 125 lines is very low, past studies have demonstrated genetic variation in natural viral resistance in *Drosophila* is often dominated by few large effect variants ([12,14–16]; but for caveats see [42]). We performed a GWAS on each phenotype separately by fitting an individual linear model for each variant in the genome using the full data. For the titre GWAS, we included focal SNP, qPCR plate, and date of injection as linear predictors. For the mortality GWAS, we included focal SNP, sex, and a sex-by-SNP interaction as linear predictors. Models were fitted using the base R linear model function ‘lm()’. We tested the significance of the SNP and SNP-by-sex predictors with a t-test, and we obtained significance thresholds for each GWAS by permuting genotypes across phenotypes 1000 times and recording the lowest *p*-value for each pseudo-dataset.

### Confirmation of GWAS hits

We chose 19 genes identified near significant GWAS hits to further test their involvement in KV infection. For each gene, we crossed a transgenic line containing a homologous foldback hairpin under the control of the Upstream Activating Sequence (UAS) to two GAL4 lines: w*; P{UAS-3xFLAG.*dCas9*.*VPR}attP40*, *P{tubP-GAL80*^*ts*^*}10; P{tubP-GAL4}LL7/TM6B*, *Tb*^*1*^ (Bloomington line #67065; hereafter referred to as *tub-GAL4*) and w*; P{GawB}Myo31DF^*NP0001*^*/CyO; P{UAS-3xFLAG*.*dCas9*.*VPR}attP2*, *P{tubP-GAL80*^*ts*^*}2* (Bloomington line #67067; hereafter referred to as *myo31DF-GAL4*). These lines drive GAL4 expression in the entire fly and in the gut, respectively, and contain a temperature-sensitive Gal80, which is able to inhibit GAL4 at the permissive temperature (18 degrees). RNAi lines included the following genes (BDSC numbers): *Pkcdelta* (28355), *btd* (29453), dos (31766), *tll* (34329), *Atg10* (40859), *Dgk* (41944), *Cip4* (53321), *hppy* (53884), *LpR2* (54461), *CG5002* (55359), *sev* (55866), *eya* (57314), *Gprk2* (57316), *Sox21b* (60120), *CG11570* (65014), *ATPCL* (65175), *Pdcd4* (66341), *CG7248* (67231), and *yin* (67334). As a control, we crossed the genetic background of the RNAi lines (Bloomington line #36304) to the two GAL4 lines. All crosses were made at 18 degrees. After eclosion, offspring were transferred to agar vials (10 flies per vial) at the non-permissive temperature (29 degrees) for two days to facilitate silencing of candidate genes, then injected with KV. We measured titre at 5 DPI for 5 vials of each KV-infected genotype for each GAL4 driver. We used a linear mixed model to analyse log-transformed viral titre in each knockdown relative to the genetic background controls, with GAL4 driver as a fixed effect, gene knockdown as a random effect, and with separate error variances for each GAL4 driver. If the random effect associated with a candidate gene was significantly different from zero, we concluded this gene played a role in determining the outcome of infection by KV. The specification of gene as a ‘random effect’ allows comparison of each knockdown to all other knockdowns, accounting for any possible overall effect of overexpressing a dsRNA hairpin. As a proof of principle, we confirmed knock-down of the largest-effect gene (*Cip4*) using the DRSC FlyPrimerBank qPCR primers *Cip4_PP33370F* (ATTGCGGGAGTGACGCTTC) and *Cip4_PP33370R* (CTGTGTGGTGAGGTTCTGCTG). We did not assess knockdown efficiency for the other crosses, and any negative findings should be treated with caution.

### Sample preparation for RNA-sequencing

We next aimed to characterise the host expression response to KV infection, and whether this differed between males and females. We injected 6 vials of 10 flies for each sex with either the control gradient solution or with KV. After 3 DPI, we homogenised flies in Trizol, extracted total nucleic acid, and enriched the sample for mRNA through DNAse treatment and poly-A selection. We used the NEB Next Ultra Directional RNA Library Prep Kit to make strand-specific paired-end libraries for each sample, following manufacturer’s instructions. Libraries were pooled and sequenced by Edinburgh Genomics (Edinburgh, UK) using three lanes of an Illumina HiSeq 4000 platform with strand-specific 75 nucleotide paired end reads. We subsequently identified a low level of Drosophila A Virus (DAV) contamination in both KV treated and untreated flies, reflecting the widespread occurrence of this virus in fly stocks and cell cultures. All reads have been submitted to the European Nucleotide Archive under project accession ERP023609.

We used SPAdes genome assembler (v3.11.1; [43]) to assemble the KV genome from RNA-sequencing reads, using the previously published genome (NC_033829.1) as an ‘untrusted contig’ (File S1).

### Differential expression analysis

We removed known sequence contaminants (primer and adapter sequences) from the paired end reads with cutadapt (V1.8.1; [44]) and mapped remaining reads to the *D*. *melanogaster* genome (FlyBase release r6.15) and to all known *Drosophila* virus genomes using STAR (V2.5.3a; [45]), with a maximum intron size of 100 KB, but otherwise default settings. We counted the number of reads mapping to each gene using the featurecounts command in the subread package (V1.5.2; [46]) and used these raw count data as input to DESeq2 (V1.16.0; [47]) for differential expression analysis. DESeq2 fits a generalised linear model for each gene, where read counts are modelled as a negative binomially distributed variable [47,48] and includes a sample-specific size factor and a dispersion parameter that depends on the shared variance of read counts for genes expressed at similar levels [47,48]. Our design matrix included sex, KV infection status, and the interaction between the two, allowing us to test for expression changes following KV infection and how these changes differ between the sexes. To account for the unintended presence of DAV, and differences in the level of DAV within and between the treatments, we also include the relative titre of DAV as a continuous predictor. Using this model, we calculated log_2_ fold changes in DESeq2, and tested for significance using Wald tests. We used the ‘plotPCA’ function implemented in DESeq2 to perform principal component analysis of the rlog-transformed read count data [47].

### GO term and network analysis

We performed five independent gene ontology (GO) term enrichment analysis, using: (1) genes with significant SNPs in the GWAS for titre; (2) genes with significant SNPs in the GWAS for mortality, (3) genes upregulated in either sex (p < 0.001); (4) genes downregulated in either sex (p < 0.001); and (5), genes significantly different between males and females (p < 0.05). For each of these gene lists, we tested for GO term enrichment using the ‘goseq’ R package (V1.26.0; [49]), which accounts for the difference in power for detecting differential expression caused by gene length, and tests for significant over-representation of genes in a GO term.

We performed a network analysis on genes identified in GWAS or RNA-sequencing studies at a liberal significance threshold (p < 0.10) to infer broadly acting pathways involved in KV infection that may have been overlooked in individual gene analyses. We used the PPI-spider tool [50] available on the bioprofiling webserver [51], which uses the IntAct database to find enriched subpathways within a provided gene list, allowing one gene absent from the provided list to mediate an interaction. Enriched pathways in the given gene list are then compared to random gene lists of the same length to assess significance.

## Results and discussion

### Isolation of Kallithea virus

**Fig 1:**
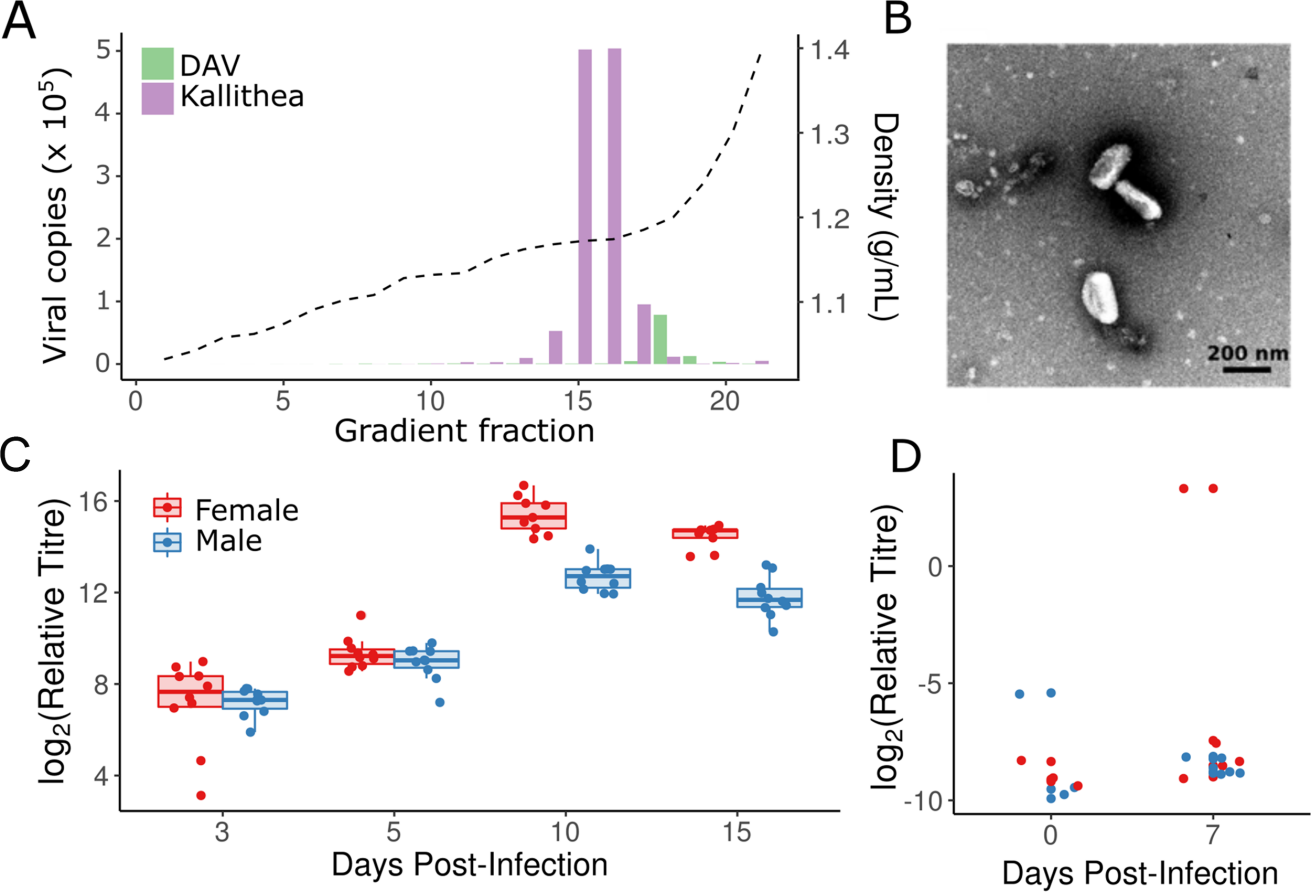
Isolation of KV and growth in flies. (A) Density gradient and virus titre: Kallithea virus (purple) was effectively separated from DAV (green) at 1.18 g/mL (dotted line) in fractions 15 and 16 of an iodixanol gradient. (B) Transmission electron micrograph of KV-positive fractions showed KV to be a rod-shaped enveloped particle, as has been described previously for other nudiviruses [55]. We did not observe unenveloped KV particles, bacteria, or RNA viruses in the isolate. (C) Relative viral titres normalised by the number of fly genomic copies and virus levels at time zero in each sex. Each point represents a vial of 10 flies. Viral titres peaked at 10 days post-infection, and were generally higher in females (red) than males (blue) late in infection. (D) We were able to infect adult flies orally by applying the viral isolate to *Drosophila* medium, although relative copy number of the virus was very low and infection was inefficient, with only 2 of 16 vials (each of 10 flies) having increased titre after one week, indicating an infectious rate lower bound of ~1% at 5x10^3^ ID50.

We isolated Kallithea Virus (KV) by gradient centrifugation following 4 rounds of serial passage in flies. Many laboratory fly stocks and cell culture lines are persistently infected with RNA viruses [17; 31], and following serial passage we identified co-infections of DAV, Nora Virus, and Drosophila C Virus (DCV) by PCR. The high prevalence of these viruses in laboratory stocks presents a substantial hurdle in the isolation of new *Drosophila* viruses, requiring the separation of the new viruses of interest. Although this can be relatively simple (e.g. separating enveloped from non-enveloped viruses), most of the recently identified *Drosophila* viruses [31,52,53] are from ssRNA virus families with buoyant densities similar to common laboratory infections. To exclude these from our isolate, we concentrated KV using a 1.18 g/mL cushion, retaining KV at the interphase, but excluding most of the contaminating RNA viruses. Subsequent equilibrium density gradient centrifugation produced a KV band at 1.17 g/mL, and with some DAV contamination at approximately 1.20 g/mL (Fig 1A). Although nudiviruses have not previously been prepared using an iodixanol gradient, the equilibrium buoyant density was consistent with the lower buoyant densities of enveloped particles [54] and similar to other enveloped dsDNA viruses (e.g. Herpesviruses: 1.15 g/mL). KV was estimated to be an approximately 650-fold higher concentration than DAV at 1.17 g/mL, and we were unable to identify intact DAV particles by electron microscopy (KV shown in Fig 1B). KV is morphologically similar to Oryctes rhinoceros nudivirus [55], with an enveloped rod-shaped virion approximately 200 nm long and 50 nm wide.

### Kallithea virus growth in flies

We injected the KV isolate into *Drosophila Oregon R* males and females, with and without *Wolbachia*, and measured viral titre at four time-points by qPCR. In females, KV increased approximately 45,000-fold by day 10, and then began to decrease (Fig 1C). In males, the KV growth pattern was altered, growing more slowly (or possibly peaking at an earlier un-sampled time point), resulting in a 7-fold lower titre than in females after 10–15 days, (nominal MCMC p-value derived from posterior samples, MCMCp = 0.002). *Wolbachia* did not affect virus growth rate in either sex (MCMCp = 0.552, S1 Fig), reaffirming previous findings that *Wolbachia* do not offer the same protection against DNA viruses in *Drosophila* as they do against RNA viruses [56].

Nudiviruses have previously been reported to spread through sexual and faecal-oral transmission routes. The *Drosophila innubila* Nudivirus (DiNV), a close relative of KV, is thought to spread faecal-orally, so we tested whether KV can spread through infected food. We found that although oral transmission occurred, it was relatively inefficient (Fig 1D). However, the concentration of DiNV found in *D*. *innubila* faeces is broadly similar to our KV isolate after gradient centrifugation ([19]; Fig 1D), but the administered suspension had been diluted 50-fold and may consequently provide a lower dose than flies encounter naturally. To explore the potential for transovarial vertical transmission or gonad-specific infections following sexual transmission (as reported for Helicoverpa nudivirus 2; [57]), we also performed qPCR on dissected ovaries and the remaining carcasses at 3 DPI (S2 Fig). We found that KV was highly enriched in the carcass relative to the ovaries. Although intra-abdominal injection could influence KV tissue-specificity, there were still substantial levels of KV in the ovaries, indicating there is not a complete barrier to infection. These results imply that KV is likely transmitted faecal-orally, as are closely related nudiviruses, but explicit tests for transovarial or sexual transmission are required.

### Sex-specific mortality, lethargy, and altered fecundity patterns following KV infection

**Fig 2:**
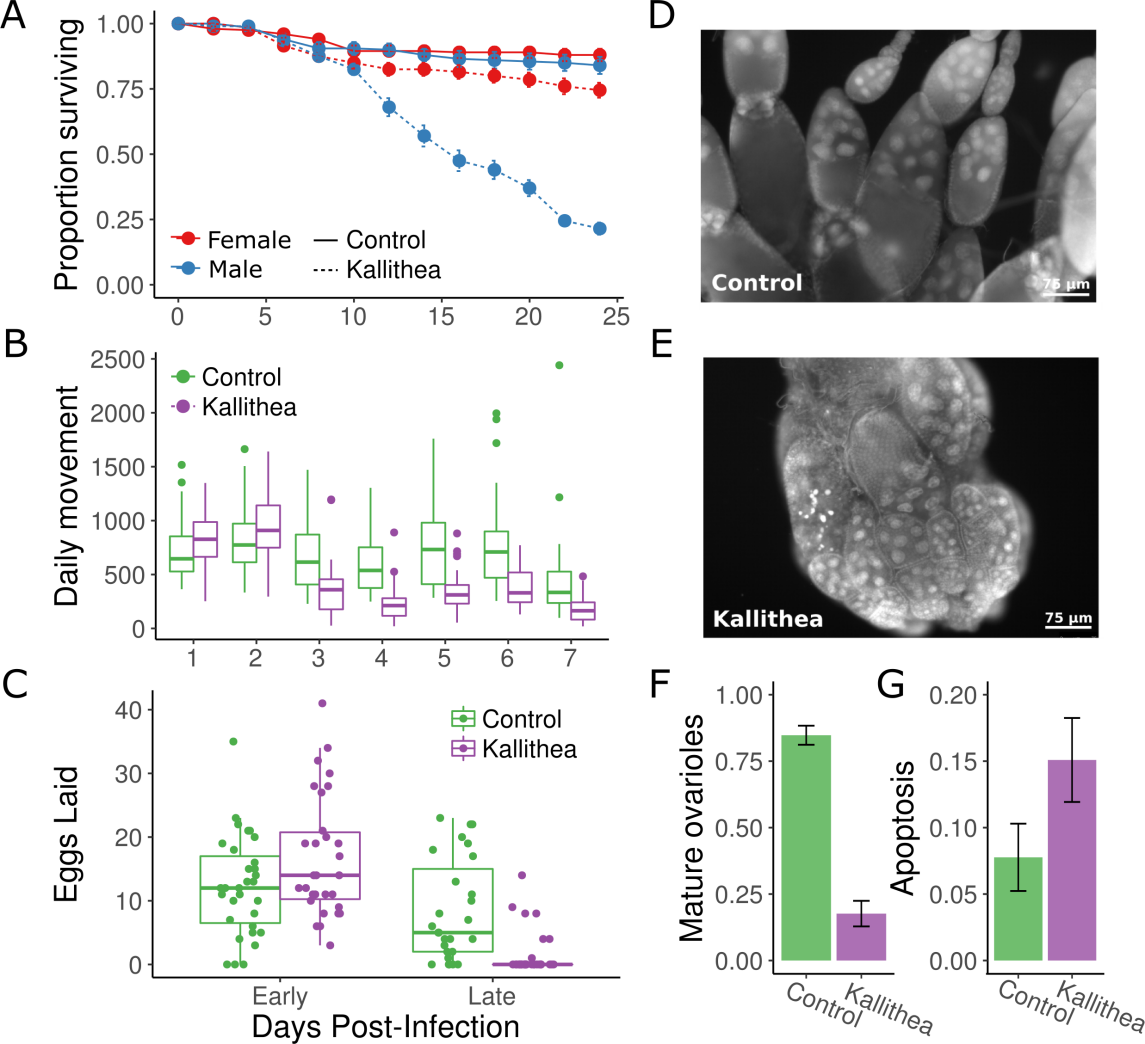
KV causes male-biased mortality, increased lethargy, and decreased fecundity. (A) Injection of KV virus into *OreR* flies led to sex-specific mortality. Infected females (red dotted line) experienced a small but significant increase in mortality, but males (blue dotted line) experienced a significantly larger rate of mortality after day 10. Flies injected with control gradient solution were unaffected (solid lines). Each point is the mean and standard error for the proportion of flies alive in each vial (10 vials of 10 flies). (B) Although females remained alive for longer, they were more lethargic. We assessed daily movement of flies injected with either chloroform-inactivated KV (green) or active KV (purple). KV-infected flies moved less from days 3–7 post-infection. (C) Females also displayed altered egg laying behaviour. Thirty pairs of flies were injected with inactive chloroform treated KV (green) or active KV (purple). KV-infected flies laid a slightly, but not significantly, higher number of eggs during early infection (1 and 2 DPI) but laid significantly fewer eggs in late infection (7 and 8 DPI). This reduction in egg laying is due to a shutdown of oogenesis before vitellogenesis (D, E), and ovaries from KV-infected flies house a lower proportion of ovarioles that include late-stage and mature egg chambers (F) and a higher proportion which contain apoptotic nurse cells (G). Ovaries were analysed 10 DPI, and error bars (F,G) show the standard error.

*Drosophila innubila* infected with DiNV suffer fitness costs including increased mortality and decreased fecundity [19]. We investigated KV-induced mortality in *D*. *melanogaster* by injecting males and females, with and without *Wolbachia*, with either control gradient solution or KV. We found that KV caused slightly, but significantly, increased mortality in females compared with controls (21% dead by day 20, vs. 11% in controls, MCMCp = 0.001), but caused a dramatically increased mortality in males compared to females (63% dead by day 20, vs. 14% in controls, sex:virus interaction MCMCp < 0.0001; Fig 2A). Therefore, males appear less tolerant of infection by KV, displaying increased mortality and a lower titre than females. We confirmed the KV-induced male death was not caused by DAV or other unknown small unenveloped RNA viruses present in our initial isolate, as chloroform treatment of the KV isolate eliminated treatment associated mortality (S3 Fig). Male-specific costs of infection are widespread across animal hosts and their pathogens (e.g. [58]), and reduced male tolerance has been found in flies infected with DCV [59]. We found that *Wolbachia* infection had no detectable effect on KV-induced mortality in males or females, and thus does not affect tolerance (MCMCp = 0.20; S1 Fig). This is consistent with previous studies showing that *Wolbachia* infection affects resistance and tolerance to RNA viruses but not a DNA virus [56].

We next tested whether female flies suffer sub-lethal fitness costs, by monitoring fly movement for a week following infection. KV-infected female flies showed similar movement patterns to chloroform-treated KV-injected flies for two days post-infection, but from three days post-infection moved significantly less (~70% reduction relative to controls; MCMCp < 0.001; Fig 2B). We conclude that females suffer from increased lethargy resulting from KV infection. In a natural setting, this could translate into fitness costs associated with increased predation, and reduced egg dispersal, mating, and foraging.

Finally, we tested whether KV infection resulted in decreased fecundity by monitoring the number of eggs laid for 8 days post-infection. We found that infected females exhibited markedly different egg laying patterns (MCMCp < 0.001; Fig 2), with KV-infected flies consistently laying fewer eggs between 7 and 8 days post-inoculation. This reduction in egg-laying during late infection could be due to a behavioural response or a cessation of oogenesis. To differentiate between these possibilities, we dissected ovaries, and determined the proportion of ovarioles that contained mature egg chambers. We found that ovaries from KV-infected flies halt oogenesis around stage 8 (MCMCp < 0.001), before vitellogenesis, and house an increased number of apoptotic egg chambers (MCMCp < 0.001) (Fig 2). This phenotype is similar to that seen upon starvation [60], and could be the manifestation of a trade-off to reroute resources to fighting infection, or of sickness-induced anorexia (e.g. [61]). Alternatively, this could be a direct consequence of viral infection, consistent with the gonadal atrophy reported for HzNV-2 [57]. Future studies should address whether this phenotype is a direct or indirect consequence of infection, and if the latter, whether it is orchestrated by the host or the virus.

### Variation in titre and mortality following KV infection

The DGRP [37] have previously been used to dissect genetic variation underlying resistance and tolerance to bacterial, fungal, and viral pathogens [12,13,62,63]. We infected 125 DGRP lines with KV and estimated broad-sense heritabilities (H^2^: the proportion of phenotypic variance attributable to genetic line) and coefficients of genetic variation (CV_G_: a mean-standardised measure of genetic variation) in viral titre and LT50 values in females, and LT50 values in males (Table 1). Our estimates of H^2^ and CV_G_ fall within the range found for resistance to other pathogens in the DGRP, although direct comparison is difficult as studies are inconsistent in the statistics used to report genetic variation. H^2^ in survival following infection with an opportunistic bacterium or fungus was similar to our estimate for survival following KV infection (*Pseudomonas aeruginosa*: H^2^ in males = 0.47, H^2^ in females = 0.38; *Metarhizium anisopliae*: H^2^ in males = 0.23, H^2^ in females = 0.27; 13), although comparing heritability can be easily confounded by differences in environmental (residual) variance [41]. Genetic variation in resistance has also been measured in response to two non-native *D*. *melanogaster* viruses (Flock House Virus and *Drosophila affinis* Sigma Virus) and two native viruses (DCV and DmelSV) in females of the DGRP. Of these, the lowest heritabilities are those associated with resistance to non-native fly viruses (FHV: narrow sense heritability h^2^ = 0.07, CV_G_ = 7; *D*. *affinis* sigma virus: h^2^ = 0.13), and the highest are associated with native fly viruses (DCV: h^2^ = 0.34, CV_G_ = 20; DmelSV: h^2^ = 0.29). Although Magwire et al [12] inferred h^2^ as half V_G_ and accounted for the homozygosity of inbred lines when inferring CV_G_, it is clear that V_G_ for resistance to KV is closer to the V_G_ for resistance to other native fly viruses than to non-native ones, at least for survival. It is also notable that CV_G_ estimates for survival are higher than estimates for titre, consistent with the observation that traits more closely related to fitness are expected to have higher CV_G_ values [41].

**Table 1:** Trait means, genetic variance (V_G_), total phenotypic variance (V_P_), heritability (H^2^), and coefficient of genetic variation (CV_G_) in titre and mortality following KV infection in the DGRP.

Trait	Trait mean	V_G_	V_P_	H^2^	CV_G_
**Titre**	1.96 [1.31–2.63]	0.05 [0.03–0.09]	0.28 [0.24–0.32]	0.19 [0.1–0.29]	11.8 [8.4–15.2]
**LT**_**50**_ **F**	25.8 [22.6–29.0]	25.2 [17.4–33.8]	44 [35.7–52.6]	0.57 [0.47–0.67]	19.5 [16.2–22.6]
**LT**_**50**_ **M**	19.6 [16.4–22.7]	10.9 [5.9–16.5]	35.7 [30.1–41.7]	0.3 [0.18–0.42]	16.7 [12.7–21]

**Fig 3:**
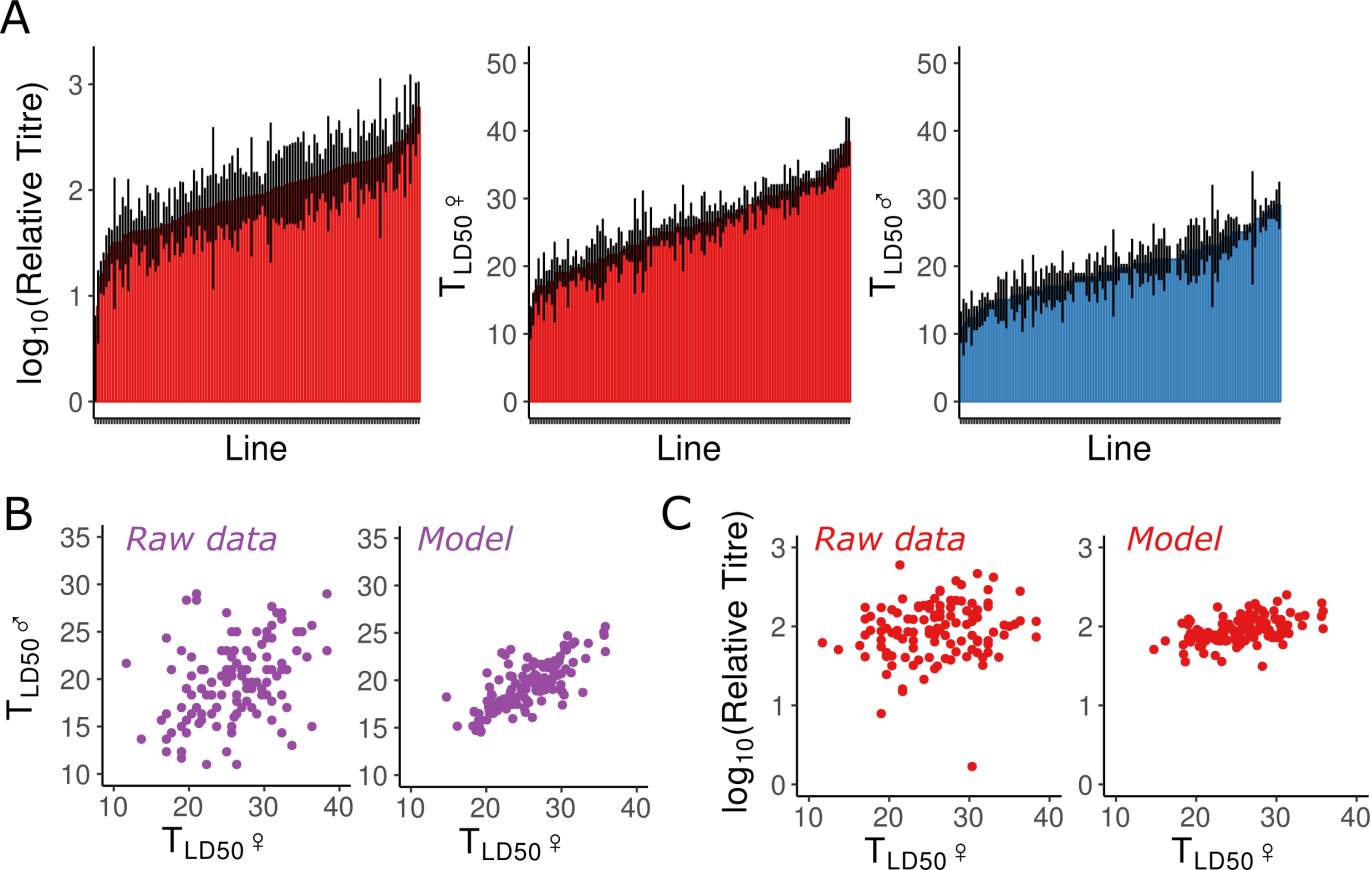
Genetic variation in resistance to KV. (A) We measured LT50 in both sexes, and titre in females, following KV injection in the DGRP. For titre, each bar represents the mean (and standard error) titre relative to fly genome copy-number, as assessed by qPCR for 5 vials of 10 flies for each of 125 DGRP lines. For LT50, each bar represents the mean time until half the flies (in a vial of 10) were dead, for three vials per line, per sex. (B, C) We used a multi-response linear mixed model to calculate genetic correlation between the traits. Shown are the raw data (left), and the estimated line effects (right) after accounting for any injection date and qPCR plate effects, and for the estimated variance among lines. Each point is a DGRP line measured for both phenotypes. We find a strong positive correlation between male and female LT50 values (B). We also observe a weak positive correlation between titre and LT50 (C).

We calculated genetic correlations between male and female mortality, and between viral titre and mortality in females (Fig 3). Note that we found no correlation between survival time following KV infection and published estimates of longevity in the absence of infection, nor to resistance to any other RNA viruses [12,64]. We found a strong positive correlation between males and females in median survival time following KV infection (0.57 [0.34–0.78]; MCMCp <0.001), such that lines in which infected males die quickly are also lines in which infected females die quickly, suggesting a shared genetic basis for early lethality following infection. We also surprisingly find a positive genetic correlation between viral titre and LT50 values (r = 0.32 [0.05–0.59], MCMCp = 0.017), such that fly lines that achieved higher titres on day 8 tended to live slightly longer. However, the effect size is small (a doubling of viral titre led to a half-day increase in median survival time) and the result is only marginally significant. The absence of a negative correlation is counter-intuitive, and contrasts with infection of the DGRP with *Providencia rettgeri* and *Metarhizium anisopliae*, and infection across *Drosophila* species with DCV, where fly lines or species with higher parasite loads suffer increased mortality [13,63,65]. This apparent decoupling of titre and mortality could result from inherent costs associated with the induction of an immune response, whereby flies that raise a more potent immune response keep KV at lower titres but induce greater tissue damage.

### Identification of candidate genes underlying host variation in KV titre

**Fig 4:**
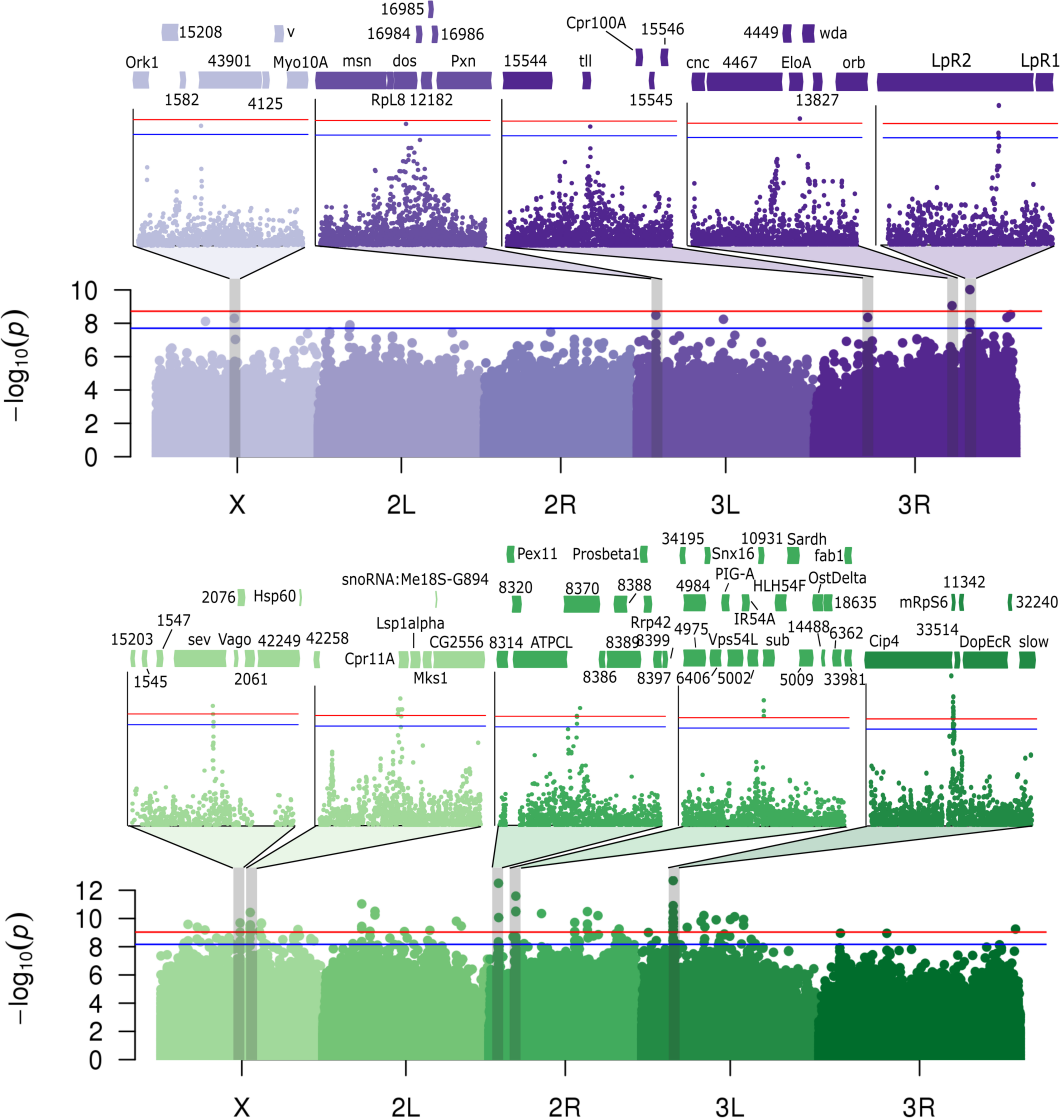
Genome-wide association of polymorphism in the DGRP with KV-induced titre and mortality. Manhattan plots showing the p-value for the effect of each polymorphism on viral titre (purple) and mortality (green). The top SNPs for each phenotype are shown in expanded inset panels, including surrounding genes. For clarity “CG” is omitted from gene identifiers. Horizontal lines show significance thresholds obtained through randomisation (p_rand_ = 0.05 in blue; p_rand_ = 0.01 in red).

Using the phenotypes in the DGRP lines measured above, we performed a genome-wide association study to identify candidate genes underlying variation in titre, LT50, and differences in LT50 between the sexes. We found 10 SNPs (9 near genes) that were significantly associated with viral titre (p_rand_ < 0.05, based on 1000 random permutations of phenotypes across lines; Fig 4). The SNP with the smallest p-value appeared in *Lipophorin receptor 2* (*LpR2*), which encodes a low-density lipoprotein receptor, previously found to be broadly required for flavivirus and rhabdovirus cell entry (e.g. [66–68]).

We tested whether these candidate polymorphisms were enriched in any molecular, biological, or cellular processes using a GO enrichment analysis, and found the top hit to be the torso signalling pathway with 2 genes of 34 in the category (p = 0.0004), *tailless* and *daughter of sevenless* (*dos*). Torso signalling is upstream of extracellular-signal-regulated kinase (ERK) pathway activation in some tissues, and human orthologues of *dos* (GAB1/GAB2/GAB4) are cleaved by an enterovirus-encoded protease, thereby activating ERK signalling and promoting viral replication [69,70]. ERK signalling is also an important regulator of virus replication in the fly midgut, where it couples nutrient availability with antiviral activity [71,72]. See S1 Table for a list of all nominally significant SNPs with associated locations, mutation types (e.g. intronic, synonymous coding, etc), nearby genes, p-values, effect sizes, and GO terms.

### Identification of candidate genes underlying host variation in KV-induced mortality

We found 86 SNPs (65 near genes) that were significantly associated with LT50 following KV infection in the DGRP (p_rand_ < 0.05; Fig 4 and S1 Table), none of which were identified in the GWAS for viral titre. We performed a GO enrichment analysis, and found genes associated with these SNPs were enriched for hydrolase activity (top molecular function GO term, p = 0.0004), stem cell fate determination (top biological process GO term, p = 0.002), and in the plasma membrane (top cell component GO term, p = 0.004), among others (S2 Table). Of these 86 SNPs, we found 34 (26 near genes) that were highly significant, and selected these for further analysis and confirmation (p_rand_ < 0.01; see S1 Table for all significant SNPs). The polymorphism with the most confident association was located in *Cdc42-interacting protein 4* (*Cip4*), a gene involved in membrane remodelling and endocytosis [73,74]. This SNP is intronic in the majority of *Cip4* transcripts, but represents a nonsynonymous polymorphism segregating leucine and proline in the first exon of *Cip4-PB* and *Cip4-PD* isoforms, perhaps indicating spliceform-specific effects on KV-induced mortality. Of particular interest from the remaining 33 highly significant SNPs was a synonymous SNP in the receptor tyrosine kinase, *sevenless*, known to interact with *dos* (above), and seven genes (*Dgk*, *Atg10*, *ATPCL*, *Hppy*, *Pkcdelta*, *Gprk2*, *Pdcd4*) previously implicated in viral pathogenesis or general immune processes. Of these, three (*Gprk4*, *hppy*, *Pkcdelta*) are involved in NF-κB signalling [75–77]. *ATPCL* was identified in an RNAi screen for factors regulating Chikungunya virus replication in humans [78] and is involved in the late replication complexes of Semliki Forest Virus [79]. Finally, *Atg10* and *Pdcd4* are involved in autophagy and apoptosis, respectively, both broadly antiviral cellular functions known to have a role in antiviral immunity in *Drosophila* [80,81]. We found no SNPs significantly associated with sex-specific KV-induced mortality (S4 Fig).

### Confirmation of GWAS hits

**Fig 5:**
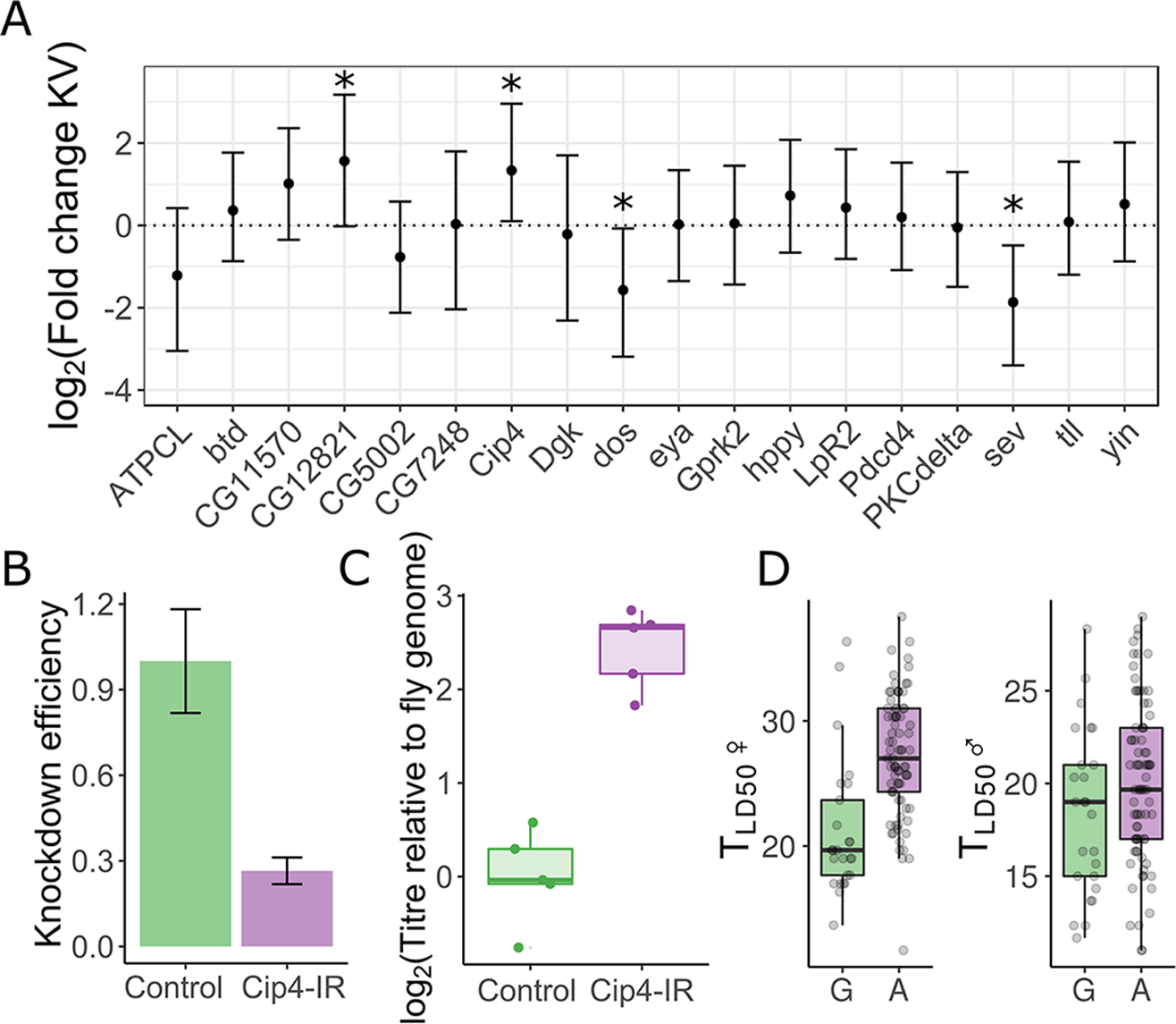
Confirmation of antiviral genes identified in GWAS. KV titre was measured in flies expressing a foldback hairpin targeting 18 genes identified in the GWAS, using GAL4 lines that knock each down in either the whole fly or specifically in the gut. (A) The data were used to estimate random effects associated with each gene knock down, plotted with 95% highest posterior density intervals. (B) Knock-down of the most confident association in the GWAS, *Cip4*, caused reduced *Cip4* RNA levels and (C) increased viral titre. (D) The associated variant (3L_4363810_SNP), was polymorphic (G/A), representing a nonsynonymous polymorphism in some splice variants, and survival following KV infection was significantly increased in fly lines with the “A” genotype, especially in females. Each point in comparison of survival in the two genotypes is a line mean. (*MCMCp < 0.05).

We chose 18 GWAS-candidate genes with available UAS-driven RNAi constructs to verify their involvement in KV infection. We found that knockdown of *Cip4* and *CG12821* caused significantly increased viral titre, and knockdown of *sev* and *dos* resulted in significantly decreased viral titre, relative to other knockdown lines (Figs 5 and S5). We confirmed *tub-GAL4>Cip4*^*IR*^ flies had reduced (26% of wild-type) *Cip4* RNA levels and a concomitant increase in viral titre relative to the genetic background control (3.4-fold increase, 95% C.I. 1.3–9.6 fold) (Fig 5). This strongly suggests that *Cip4* is a KV restriction factor that likely segregates for functional polymorphism affecting survival following KV infection (Fig 5). It is known that baculovirus budded virions enter cells through clathrin-mediated endocytosis or micropinocytosis [82,83], and gain their envelope at the cell membrane upon exit [84]. Cip4 could therefore plausibly enact an antiviral effect by limiting KV cell entry or spread, perhaps through its known function in cell membrane remodelling and trafficking.

### Differential expression following KV infection

**Fig 6:**
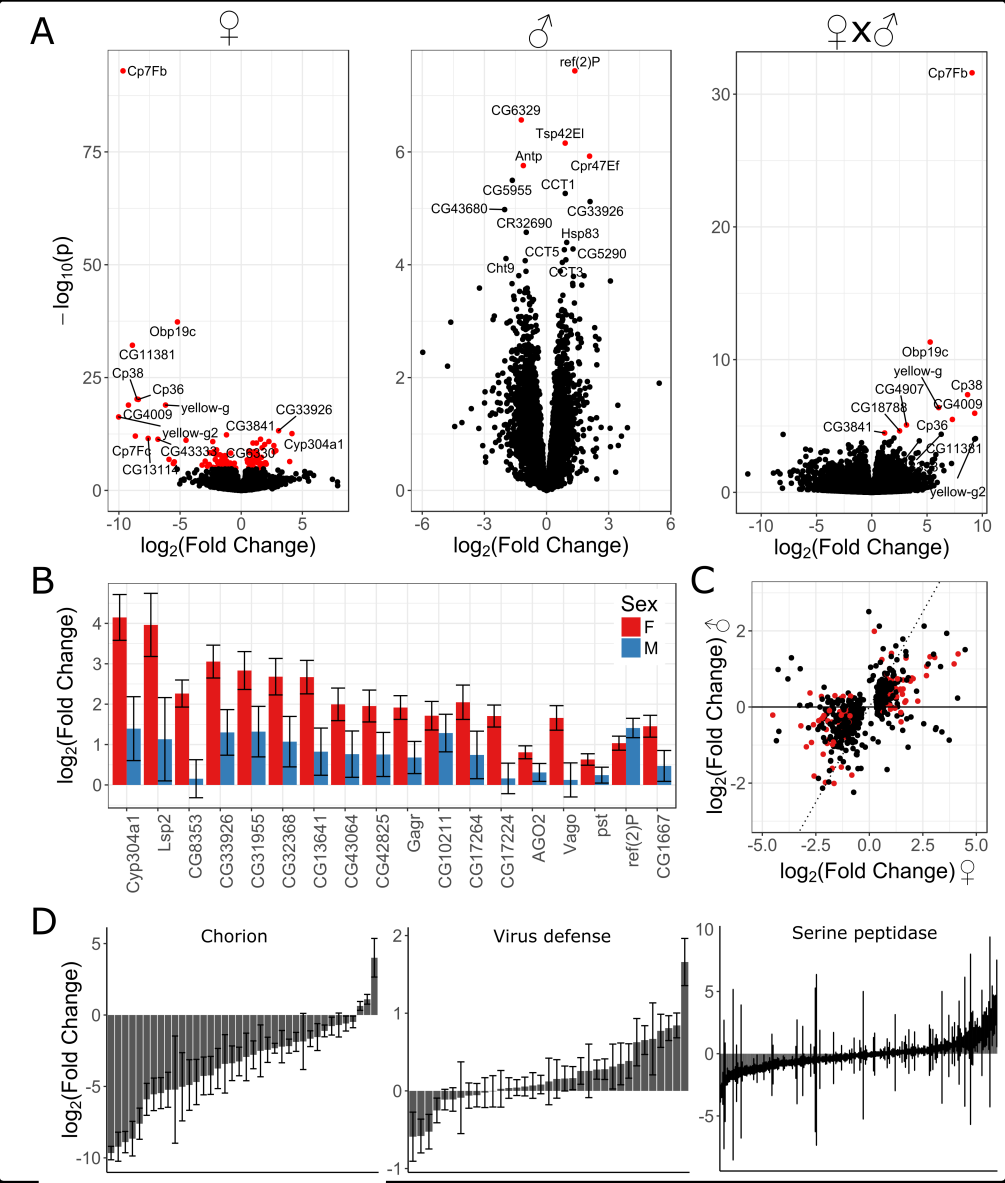
KV induces differential regulation of chorion, virus defense, and serine endopeptidase genes. (A) Volcano plots showing fold changes and p-values from Wald tests for differential expression of *D*. *melanogaster* genes following KV infection for females (left), males (center), those different between the sexes (right), with DAV read count fit as a covariate and nominal significance threshold of p < 0.001. In each panel, the genes with the smallest p-values are labelled. All of the genes that were significantly differentially regulated between the sexes are highly significant in females. (B) Highly induced genes are mostly functionally unannotated, but include some with known roles in viral pathogenesis. (C) The male response is correlated with females, but muted, with few genes identified as significantly differentially expressed in males. Genes with weak evidence of differential expression in either sex (p < 0.05) are plotted, where the dotted line represents a perfect correlation, and red points are genes identified as significantly differentially expressed. (D) The top GO enrichment terms for each GO class (Molecular Function, Biological Process, Cellular Component) were genes involved the chorion, virus defense, and serine peptidase activity. For each plot, estimated fold changes and their associated standard errors are plotted for every gene matching the GO term, regardless of the significance of the Wald test. Generally, chorion genes were downregulated, virus defense genes were upregulated, and serine peptidases were downregulated.

Previous transcriptional profiling in response to RNA virus infection has shown upregulation of heatshock proteins, JAK-STAT, JNK, and Imd pathways [4,28,85,86]. However, the *D*. *melanogaster* expression response to a DNA virus has not previously been investigated. We separately injected male and female flies with control gradient solution or KV and extracted mRNA for sequencing 3 days post-infection. KV gene expression increased dramatically 3 days post-inoculation, consistent with our qPCR analysis of genome copy-number (Figs 1 and S10). Although not previously detectable by PCR, RNAseq read mapping identified a low level of DAV in both control and KV-infected flies, with an overall higher level in KV-infected flies. To account for this potentially confounding contaminant, we fitted the number of DAV-mapped reads as a covariate in the differential expression analysis, and used a stringent Benjamini–Hochberg adjusted significance threshold of *p* < 0.001 to infer nominal significance. We found 54 genes upregulated and 79 genes downregulated in response to KV in either males or females (Fig 6 and S3 Table). There was no enrichment for GWAS hits among the KV-responsive genes (S6 Fig). Principal components analysis on depth-normalised read counts separated males and females along PC1 and partially separated KV-infected and control-injected libraries along PC3 (S7 Fig). GO term analysis identified ‘defense response to virus’ (p = 3.1x10^-4^), ‘serine peptidase activity’ (p = 1.2x10^-7^, identified in part due to downregulation of Jonah family serine proteases), and ‘chorion’ (p < 1x10^-8^) as the most highly enriched biological process, molecular function, and cellular component, respectively (Fig 6 and S4 Table). Subsequent network analysis identified a large pathway of interactions enriched for genes either differentially expressed or associated with variation in KV infection, including known defense response genes (S8 Fig).

There are few described induced antiviral immune effectors in *Drosophila* (e.g. [81]). In line with this we observe 57% of differentially expressed genes have not yet been named (i.e. “CG” genes), significantly greater than the genome-wide rate of 41% (p = 3x10^-4^), and the most highly induced genes have not been implicated in viral pathogenesis. The cytochrome P450 family gene *Cyp304a1* was most highly upregulated, concomitant with the upregulation of four other genes in this family (*Cyp309a1*, *Cyp309a2*, *Cyp4p3*, and *Cyp6a20*). The next most highly induced genes include the hemocyanin *Larval serum protein 2*, the cytidine deaminase *CG8353*, four genes without functional annotation or recognisable domains (*CG33926*, *CG31955*, *CG32368*, *CG13641*), and an additional six genes without functional annotation (*CG43064*, *CG42825*, *Gagr*, *CG10211*, *CG17264*, and *CG17224* –the last two of which are adjacent on chromosome arm 2L). We also note the striking but variable upregulation of 11 of the 24 Tweedle genes (S9 Fig) in some (but not all) of the infected samples. These are secreted, insect-specific cuticle proteins that regulate body shape [87], and are also upregulated in response to Sindbis virus infection in cell culture [88], perhaps suggesting a general role in viral pathogenesis.

Genes with known involvement in viral pathogenesis were also found to be induced following KV infection. The RNAi effector *AGO2* was upregulated, consistent with the previous results that DNA viruses are a target of the RNAi pathway [21,28,29]. *Vago*, an antiviral factor downstream of Dicer-2 [89], was upregulated and was also adjacent to a SNP found in the mortality GWAS (*dos*; Figs 4 and 6), as were *pastrel* and *ref(2)P*, identified in previous genome wide association analyses for resistance to DCV and DMelSV, respectively. Finally, we found that KV induced expression of *CG1667*, the *Drosophila* homologue of STING. The vertebrate cGAS-STING pathway is involved in cytosolic DNA sensing and activation of immune factors in response to DNA virus infection [90]. This upregulation of *CG1667* may suggest that this is another pathway conserved between *Drosophila* and vertebrates.

As we had observed male and female differences in KV-induced mortality and titre (Figs 1 and 2), we tested for sex-specific transcriptional regulation in response to KV infection. We found that females and males had similar patterns of differential expression following KV infection (spearman rank correlation coefficient, ρ = 0.57, p = 2.2x10^-16^), although the male response was often less potent (Fig 6). Nine genes were significantly differentially expressed (p < 0.05) between the sexes specifically in response to KV (S3 Table), and these were all downregulated in females and highly enriched for genes associated with the chorion (Fig 6 and S4 Table). Strikingly, all but three genes classified with the GO term ‘chorion’ were downregulated in females (Fig 6), consistent with the observed reduction in mature ovarioles and eggs during late infection, and implying a substantial reorganization of oogenesis (Fig 2). We did not identify any previously described immune genes with significant sex-specific regulation during KV infection.

## Conclusions

We have isolated Kallithea virus, a dsDNA nudivirus that naturally infects *D*. *melanogaster*, and find it to be experimentally tractable. KV infection leads to reduced fertility and movement in females, highlighting the importance of measuring fitness associated traits besides longevity. Although males suffered greater mortality than females, they achieved lower titres, consistent with increased resistance and/or reduced tolerance in males. Similar to RNA viruses, we identified moderate host genetic variation in resistance to KV infection, however, we found that the underlying genetic architecture of this variation is unlike previously studied RNA viruses of *D*. *melanogaster*, in which a high proportion of genetic variation was apparently determined by a small number of loci. This could reflect a difference in the co-evolutionary dynamics between *D*. *melanogaster* and KV, versus other RNA viruses such as DCV and DMelSV. The *D*. *melanogaster* transcriptional response to KV included genes with known involvement in viral pathogenesis, but also genes that could represent infection responses distinctive to DNA viruses or KV, including downregulation of chorion genes. Upregulation of widely conserved immune factors, such as *STING*, represent promising candidates involved in fly antiviral immunity, and demonstrate the continued utility of the *Drosophila* system for understanding host-virus interactions.

## Supporting information

S1 Fig No effect of *Wolbachia* on KV growth or KV-induced mortality. Upper panels: Log-transformed relative viral titre in *Wolbachia* positive (green) or negative (orange) *OreR* female and male flies. Lower panels: mortality curves for gradient control-injected (circle) or KV-injected (triangle) *OreR* female and male flies, with or without Wolbachia.
(TIF)

S2 Fig KV enriched in carcass relative to ovary. Females had higher viral titres in non-ovary tissues at 3 DPI. However, this could be affected by the route of infection, and the average ploidy of each sample.
(TIF)

S3 Fig KV-induced male mortality is not caused by contaminating RNA viruses. Chloroform-treatment is expected to inactivate enveloped viruses such as KV, but unenveloped viruses (including most +ssRNA viruses) are expected to retain infectivity. We confirmed mortality following KV infection was not caused by contaminating DAV by comparing injection of the KV isolate with (green) or without (purple) inactivating chloroform treatment.
(TIF)

S4 Fig Sex-by-genotype interaction significance manhattan plot. No polymorphism had a significant effect on sex-specific mortality. The blue line denotes p_rand_ = 0.05 and the red line is p_rand_ = 0.01.
(TIF)

S5 Fig Virus titre in other confirmed GWAS knock-down lines. KV titre was measured in flies expressing a foldback hairpin targeting 18 genes identified in the GWAS, using GAL4 lines that knock each down in either the whole fly (*tub-GAL4*, green) or specifically in the gut (*myo31DF-GAL4*, purple). Only those causing a significant increase in titre relative to other knock-down lines (e.g. Fig 5) are shown here. Note that the cross between *myo31DF-GAL4* and *CG12821*^*IR*^ was inexplicably lethal, and that titre was highly variable in some of the other *myo31DF-GAL4* crosses.
(TIF)

S6 Fig No enrichment of KV infection-associated SNPs in KV-responsive genes. Genes were split into KV-responsive and KV-unresponsive genes based on the RNA sequencing differential expression analysis (p < 0.05). The largest effect size (max β^SNP^) and lowest p-value was recorded for each gene in each GWAS, and compared between the KV-responsive and unresponsive genes. We find no significant difference between any comparisons.
(TIF)

S7 Fig RNA sequencing: Library quality and verification. (A) The first three principal components of read counts per gene in RNA-sequencing data, plotted such that each library is represented by a point. Males (blue) and females (red) are separated on PC1. Control-injected (green) and KV-injected (purple) are separated on PC3. For (B), we clustered libraries based on expression of the 1000 most variable genes, where each row on the heatmap is a gene, and the columns are libraries. Together, these analyses identified two possible outlier libraries, which were excluded (black rectangles in A and B). (C) We selected 13 well-studied immune genes and genes with a clear phenotype association (e.g. chorion proteins), distributed across the range of differential expression values, for qPCR verification. Using 5 independent biological replicates from the outbred DGRP population, we confirmed that differential expression for these genes was highly correlated between qPCR and RNA-seq (r^2^ = 0.77, p = 0.002). (D) We found low-level DAV contamination in our RNA-sequencing experiment. The plot shows the relationship between DAV viral titre and average KV gene expression, where each point is the number of reads mapping to KV and DAV for each library, normalised by library size factor and genome length. Note that KV is plotted on a log_10_ scale, but DAV on a linear scale.
(TIF)

S8 Fig Pathway analysis of genes identified by differential expression analysis or GWAS. Network analysis identified a pathway marginally enriched for genes identified in these studies (p = 0.03). Shown are interactions (edges) between genes identified in GWAS or RNA-sequencing studies (square nodes), allowing a single gene not included in the original gene list to mediate an interaction (triangular nodes). Genes (nodes) are coloured by functional annotation and white nodes are unannotated. Node labels are associated with the bottom-right corners of nodes.
(TIF)

S9 Fig Variable differential expression of the Tweedle gene family. A subset of KV-infected (purple) vials showed very high expression of Tweedle genes, whereas these were mostly unexpressed in control (green) adult flies.
(TIF)

S10 Fig Expression of Kallithea virus genes. Most KV genes are expressed at 3 DPI. One KV-injected vial of flies had a lower level of infection. Control libraries also showed mapping to KV genes, most likely due to a low level (<0.5%) of barcode switching among libraries run together. The lower panel is a continuation of the upper panel.
(TIF)

S1 File Kallithea virus assembly from RNA-seq reads. (FASTA)

S1 Table Significant GWAS hits. (XLSX)

S2 Table Significant GO term enrichment for GWAS hits. (XLSX)

S3 Table Differentially expressed genes. (XLSX)

S4 Table Significant GO terms for DE genes. (XLSX)

## References

[1] Dupuis S., Jouanguy E., Al-Hajjar S., Fieschi C., Al-Mohsen I. Z. et al, 2003 Impaired response to interferon-α/β and lethal viral disease in human STAT1 deficiency. Nat. Genet. 33: 388–391. doi: 10.1038/ng1097 12590259 10.1038/ng1097

[2] Sharma S., tenOever B. R., Grandvaux N., Zhou G.-P., Lin R. et al, 2003 Triggering the Interferon Antiviral Response Through an IKK-Related Pathway. Science (80-.). 300: 1148–1151. 10.1126/science.1081315 12702806

[3] Zambon R. A., Nandakumar M., Vakharia V. N., and Wu L. P., 2005 The Toll pathway is important for an antiviral response in Drosophila. Proc. Natl. Acad. Sci. U. S. A. 102: 7257–62. doi: 10.1073/pnas.0409181102 15878994 10.1073/pnas.0409181102 PMC1129099

[4] Dostert C., Jouanguy E., Irving P., Troxler L., Galiana-Arnoux D. et al, 2005 The Jak-STAT signaling pathway is required but not sufficient for the antiviral response of drosophila. Nat. Immunol. 6: 946–953. doi: 10.1038/ni1237 16086017 10.1038/ni1237

[5] Wang X.-H., Aliyari R., Li W.-X., Li H.-W., Kim K. et al, 2006 RNA interference directs innate immunity against viruses in adult Drosophila. Science 312: 452–4. doi: 10.1126/science.1125694 16556799 10.1126/science.1125694 PMC1509097

[6] Avadhanula V., Weasner B. P., Hardy G. G., Kumar J. P., and Hardy R. W., 2009 A Novel System for the Launch of Alphavirus RNA Synthesis Reveals a Role for the Imd Pathway in Arthropod Antiviral Response (S.-W. Ding, Ed.). PLoS Pathog. 5: e1000582 doi: 10.1371/journal.ppat.1000582 19763182 10.1371/journal.ppat.1000582 PMC2738967

[7] Maillard P. V., Ciaudo C., Marchais A., Li Y., Jay F. et al, 2013 Antiviral RNA Interference in Mammalian Cells. Science (80-.). 342: 235–238. 10.1126/science.1241930 PMC3853215 24115438

[8] Li Y., Lu J., Han Y., Fan X., and Ding S.-W., 2013 RNA Interference Functions as an Antiviral Immunity Mechanism in Mammals. Science (80-.). 342: 231–234. 10.1126/science.1241911 PMC3875315 24115437

[9] Ferguson H. M., and Read A. F., 2002 Why is the effect of malaria parasites on mosquito survival still unresolved? Trends Parasitol. 18: 256–261. 12036738 10.1016/s1471-4922(02)02281-x

[10] Compton A. A., Hirsch V. M., and Emerman M., 2012 The host restriction factor APOBEC3G and retroviral Vif protein coevolve due to ongoing genetic conflict. Cell Host Microbe 11: 91–8. doi: 10.1016/j.chom.2011.11.010 22264516 10.1016/j.chom.2011.11.010 PMC3266539

[11] van Mierlo J. T., Overheul G. J., Obadia B., van Cleef K. W. R., Webster C. L. et al, 2014 Novel Drosophila Viruses Encode Host-Specific Suppressors of RNAi (D. S. Schneider, Ed.). PLoS Pathog. 10: e1004256 doi: 10.1371/journal.ppat.1004256 25032815 10.1371/journal.ppat.1004256 PMC4102588

[12] Magwire M. M., Fabian D. K., Schweyen H., Cao C., Longdon B. et al, 2012 Genome-Wide Association Studies Reveal a Simple Genetic Basis of Resistance to Naturally Coevolving Viruses in Drosophila melanogaster (M. L. Wayne, Ed.). PLoS Genet. 8: e1003057 doi: 10.1371/journal.pgen.1003057 23166512 10.1371/journal.pgen.1003057 PMC3499358

[13] Wang J. B., Lu H.-L., and St. Leger R. J., 2017 The genetic basis for variation in resistance to infection in the Drosophila melanogaster genetic reference panel (A. Andrianopoulos, Ed.). PLOS Pathog. 13: e1006260 doi: 10.1371/journal.ppat.1006260 28257468 10.1371/journal.ppat.1006260 PMC5352145

[14] Contamine D., Petitjean A. M., and Ashburner M., 1989 Genetic resistance to viral infection: the molecular cloning of a Drosophila gene that restricts infection by the rhabdovirus sigma. Genetics 123: 525–33. 2557263 10.1093/genetics/123.3.525 PMC1203824

[15] Magwire M. M., Bayer F., Webster C. L., Cao C., and Jiggins F. M., 2011 Successive Increases in the Resistance of Drosophila to Viral Infection through a Transposon Insertion Followed by a Duplication (D. J. Begun, Ed.). PLoS Genet. 7: e1002337 doi: 10.1371/journal.pgen.1002337 22028673 10.1371/journal.pgen.1002337 PMC3197678

[16] Cogni R., Cao C., Day J. P., Bridson C., and Jiggins F. M., 2016 The genetic architecture of resistance to virus infection in *Drosophila*. Mol. Ecol. 25: 5228–5241. doi: 10.1111/mec.13769 27460507 10.1111/mec.13769 PMC5082504

[17] Brun, P., and N. Plus, 1980 The viruses of Drosophila, pp. 625–702 in The genetics and biology of Drosophila.

[18] Huszar T., and Imler J.-L., 2008 Drosophila viruses and the study of antiviral host-defense. Adv. Virus Res. 72: 227–65. doi: 10.1016/S0065-3527(08)00406-5 19081493 10.1016/S0065-3527(08)00406-5

[19] Unckless R. L., 2011 A DNA Virus of Drosophila (R. DeSalle, Ed.). PLoS One 6: e26564 doi: 10.1371/journal.pone.0026564 22053195 10.1371/journal.pone.0026564 PMC3203887

[20] Xu J., and Cherry S., 2014 Viruses and antiviral immunity in Drosophila. Dev. Comp. Immunol. 42: 67–84. doi: 10.1016/j.dci.2013.05.002 23680639 10.1016/j.dci.2013.05.002 PMC3826445

[21] Bronkhorst A. W., van Cleef K. W. R., Vodovar N., Ince I. A., Blanc H. et al., 2012 The DNA virus Invertebrate iridescent virus 6 is a target of the Drosophila RNAi machinery. Proc. Natl. Acad. Sci. 109: E3604–E3613. doi: 10.1073/pnas.1207213109 23151511 10.1073/pnas.1207213109 PMC3529082

[22] Herniou E. A., Olszewski J. A., O’Reilly D. R., and Cory J. S., 2004 Ancient coevolution of baculoviruses and their insect hosts. J. Virol. 78: 3244–51. doi: 10.1128/JVI.78.7.3244-3251.2004 15016845 10.1128/JVI.78.7.3244-3251.2004 PMC371050

[23] Breitenbach J. E., Shelby K. S., and Popham H. J. R., 2011 Baculovirus Induced Transcripts in Hemocytes from the Larvae of Heliothis virescens. Viruses 3: 2047–2064. doi: 10.3390/v3112047 22163334 10.3390/v3112047 PMC3230841

[24] Noland J., Breitenbach J., Popham H., Hum-Musser S., Vogel H. et al, 2013 Gut Transcription in Helicoverpa zea is Dynamically Altered in Response to Baculovirus Infection. Insects 4: 506–520. doi: 10.3390/insects4030506 26462433 10.3390/insects4030506 PMC4553479

[25] Nguyen Q., Nielsen L. K., and Reid S., 2013 Genome scale transcriptomics of baculovirus-insect interactions. Viruses 5: 2721–47. doi: 10.3390/v5112721 24226166 10.3390/v5112721 PMC3856412

[26] McTaggart S. J., Hannah T., Bridgett S., Garbutt J. S., Kaur G. et al, 2015 Novel insights into the insect trancriptome response to a natural DNA virus. BMC Genomics 16: 310 doi: 10.1186/s12864-015-1499-z 25924671 10.1186/s12864-015-1499-z PMC4415287

[27] Williams T., 2008 Natural invertebrate hosts of iridoviruses (Iridoviridae). Neotrop. Entomol. 37: 615–632. 19169548 10.1590/s1519-566x2008000600001

[28] Kemp C., Mueller S., Goto A., Barbier V., Paro S. et al, 2013 Broad RNA interference-mediated antiviral immunity and virus-specific inducible responses in Drosophila. J. Immunol. 190: 650–8. doi: 10.4049/jimmunol.1102486 23255357 10.4049/jimmunol.1102486 PMC3538939

[29] Bronkhorst A. W., van Cleef K. W. R., Venselaar H., and van Rij R. P., 2014 A dsRNA-binding protein of a complex invertebrate DNA virus suppresses the Drosophila RNAi response. Nucleic Acids Res. 42: 12237–12248. doi: 10.1093/nar/gku910 25274730 10.1093/nar/gku910 PMC4231766

[30] Hill T., and Unckless R. L., 2018 The dynamic evolution of Drosophila innubila Nudivirus. Infect. Genet. Evol. 57: 151–157 doi: 10.1016/j.meegid.2017.11.013 29155284 10.1016/j.meegid.2017.11.013 PMC5725240

[31] Webster C. L., Waldron F. M., Robertson S., Crowson D., Ferrari G. et al, 2015 The Discovery, Distribution, and Evolution of Viruses Associated with Drosophila melanogaster. PLoS Biol. 13: e1002210 doi: 10.1371/journal.pbio.1002210 26172158 10.1371/journal.pbio.1002210 PMC4501690

[32] Kapun M., Barron Aduriz M. G., Staubach F., Vieira J., Obbard D., et al, 2018 Genomic analysis of European Drosophila melanogaster populations on a dense spatial scale reveals longitudinal population structure and continent-wide selection. *BioRxiv*. 10.1093/molbev/msaa120 PMC7475034 32413142

[33] Thézé J., Bézier A., Periquet G., Drezen J.-M., and Herniou E. A., 2011 Paleozoic origin of insect large dsDNA viruses. Proc. Natl. Acad. Sci. U. S. A. 108: 15931–5. doi: 10.1073/pnas.1105580108 21911395 10.1073/pnas.1105580108 PMC3179036

[34] Herniou E. A., Huguet E., Thézé J., Bézier A., Periquet G. et al, 2013 When parasitic wasps hijacked viruses: genomic and functional evolution of polydnaviruses. Philos. Trans. R. Soc. Lond. B. Biol. Sci. 368: 20130051 doi: 10.1098/rstb.2013.0051 23938758 10.1098/rstb.2013.0051 PMC3758193

[35] Gauthier J., Drezen J.-M., and Herniou E. A., 2017 The recurrent domestication of viruses: major evolutionary transitions in parasitic wasps. Parasitology. 23: 1–13. 10.1017/S0031182017000725 28534452

[36] Wang Y., and Jehle J. A., 2009 Nudiviruses and other large, double-stranded circular DNA viruses of invertebrates: New insights on an old topic. J. Invertebr. Pathol. 101: 187–193. doi: 10.1016/j.jip.2009.03.013 19460388 10.1016/j.jip.2009.03.013

[37] Mackay T. F. C., Richards S., Stone E. A., Barbadilla A., Ayroles J. F. et al, 2012 The Drosophila melanogaster Genetic Reference Panel. Nature 482: 173–178. doi: 10.1038/nature10811 22318601 10.1038/nature10811 PMC3683990

[38] Lee Y. S., Nakahara K., Pham J. W., Kim K., He Z. et al, 2004 Distinct Roles for Drosophila Dicer-1 and Dicer-2 in the siRNA/miRNA Silencing Pathways. Cell 117: 69–81. 15066283 10.1016/s0092-8674(04)00261-2

[39] Hadfield J. D., 2010 MCMC Methods for Multi-Response Generalized Linear Mixed Models: The MCMCglmm R Package. J. Stat. Softw. 33: 1–2220808728

[40] Pfeiffenberger C., Lear B. C., Keegan K. P., and Allada R., 2010 Locomotor activity level monitoring using the Drosophila Activity Monitoring (DAM) System. Cold Spring Harb. Protoc. 2010: pdb.prot5518. 10.1101/pdb.prot5518 21041391

[41] Houle D., 1992 Comparing evolvability and variability of quantitative traits. Genetics 130: 195–204. 1732160 10.1093/genetics/130.1.195 PMC1204793

[42] King E. G., and Long A. D., 2017 The Beavis Effect in Next-Generation Mapping Panels in Drosophila melanogaster. G3 (Bethesda). 7: 1643–1652. 28592647 10.1534/g3.117.041426 PMC5473746

[43] Bankevich A., Nurk S., Antipov D., Gurevich A.A., Dvorkin M., et al, 2012 SPAdes: a new genome assembly algorithm and its applications to single-cell sequencing. Journal of computational biology, 19(5): 455–477. doi: 10.1089/cmb.2012.0021 22506599 10.1089/cmb.2012.0021 PMC3342519

[44] Martin M., 2011 Cutadapt removes adapter sequences from high-throughput sequencing reads. EMBnet.journal 17: 10.

[45] Dobin A., Davis C. A., Schlesinger F., Drenkow J., Zaleski C. et al, 2013 STAR: ultrafast universal RNA-seq aligner. Bioinformatics 29: 15–21. doi: 10.1093/bioinformatics/bts635 23104886 10.1093/bioinformatics/bts635 PMC3530905

[46] Liao Y., Smyth G. K., and Shi W., 2013 The Subread aligner: fast, accurate and scalable read mapping by seed-and-vote. Nucleic Acids Res. 41: e108–e108. doi: 10.1093/nar/gkt214 23558742 10.1093/nar/gkt214 PMC3664803

[47] Love M. I., Huber W., and Anders S., 2014 Moderated estimation of fold change and dispersion for RNA-seq data with DESeq2. Genome Biol. 15: 550 doi: 10.1186/s13059-014-0550-8 25516281 10.1186/s13059-014-0550-8 PMC4302049

[48] Anders S., and Huber W., 2010 Differential expression analysis for sequence count data. Genome Biol. 11: R106 doi: 10.1186/gb-2010-11-10-r106 20979621 10.1186/gb-2010-11-10-r106 PMC3218662

[49] Young M. D., Wakefield M. J., Smyth G. K., and Oshlack A., 2010 Gene ontology analysis for RNA-seq: accounting for selection bias. Genome Biol. 11: R14 doi: 10.1186/gb-2010-11-2-r14 20132535 10.1186/gb-2010-11-2-r14 PMC2872874

[50] Antonov A. V., Dietmann S., Rodchenkov I., and Mewes H. W., 2009 PPI spider: A tool for the interpretation of proteomics data in the context of protein-protein interaction networks. Proteomics 9: 2740–2749. doi: 10.1002/pmic.200800612 19405022 10.1002/pmic.200800612

[51] Antonov A. V, 2011 BioProfiling.de: analytical web portal for high-throughput cell biology. Nucleic Acids Res. 39: W323–7. 10.1093/nar/gkr372 PMC3125774 21609949

[52] Webster C. L., Longdon B., Lewis S. H., and Obbard D. J., 2016 Twenty-Five New Viruses Associated with the Drosophilidae (Diptera). Evol. Bioinform. Online 12: 13–25. doi: 10.4137/EBO.S39454 27375356 10.4137/EBO.S39454 PMC4915790

[53] Medd N. C., Fellous S., Waldron F. M., Xuéreb A., Nakai M., et al, 2018 The virome of Drosophila suzukii, an invasive pest of soft fruit. Virus Evolution 4(1):vey009 doi: 10.1093/ve/vey009 29644097 10.1093/ve/vey009 PMC5888908

[54] Feng Z., Hensley L., McKnight K. L., Hu F., Madden V. et al, 2013 A pathogenic picornavirus acquires an envelope by hijacking cellular membranes. Nature 496: 367–71. doi: 10.1038/nature12029 23542590 10.1038/nature12029 PMC3631468

[55] Huger A. M., 2005 The Oryctes virus: its detection, identification, and implementation in biological control of the coconut palm rhinoceros beetle, Oryctes rhinoceros (Coleoptera: Scarabaeidae). J. Invertebr. Pathol. 89: 78–84. doi: 10.1016/j.jip.2005.02.010 16039308 10.1016/j.jip.2005.02.010

[56] Teixeira L., Ferreira A., and Ashburner M., 2008 The bacterial symbiont Wolbachia induces resistance to RNA viral infections in Drosophila melanogaster. (L. Keller, Ed.). PLoS Biol. 6: e2. 10.1371/journal.pbio.1000002 PMC2605931 19222304

[57] Burand J. P., Kim W., Afonso C. L., Tulman E. R., Kutish G. F. et al, 2012 Analysis of the Genome of the Sexually Transmitted Insect Virus Helicoverpa zea Nudivirus 2. Viruses 4: 28–61. doi: 10.3390/v4010028 22355451 10.3390/v4010028 PMC3280521

[58] Zuk M., 2009 The sicker sex. PLoS Pathog. 5: e1000267 doi: 10.1371/journal.ppat.1000267 19180235 10.1371/journal.ppat.1000267 PMC2628977

[59] Gupta V., and Vale P. F., 2017 Nonlinear disease tolerance curves reveal distinct components of host responses to viral infection. R. Soc. open Sci. 4: 170342 doi: 10.1098/rsos.170342 28791163 10.1098/rsos.170342 PMC5541558

[60] Jouandin P., Ghiglione C., and Noselli S., 2014 Starvation induces FoxO-dependent mitotic-to-endocycle switch pausing during Drosophila oogenesis. Development 141: 3013–3021. doi: 10.1242/dev.108399 24993942 10.1242/dev.108399 PMC6514422

[61] Ayres J. S., and Schneider D. S., 2009 The Role of Anorexia in Resistance and Tolerance to Infections in Drosophila (D. Promislow, Ed.). PLoS Biol. 7: e1000150 doi: 10.1371/journal.pbio.1000150 19597539 10.1371/journal.pbio.1000150 PMC2701602

[62] Bou Sleiman M. S., Osman D., Massouras A., Hoffmann A. A., Lemaitre B. et al, 2015 Genetic, molecular and physiological basis of variation in Drosophila gut immunocompetence. Nat. Commun. 6: 7829 doi: 10.1038/ncomms8829 26213329 10.1038/ncomms8829 PMC4525169

[63] Howick V. M., and Lazzaro B. P., 2017 The genetic architecture of defence as resistance to and tolerance of bacterial infection in Drosophila melanogaster. Mol. Ecol. 26: 1533–1546. doi: 10.1111/mec.14017 28099780 10.1111/mec.14017

[64] Ivanov D. K., Escott-Price V., Ziehm M., Magwire M. M., Mackay T. F. C. et al, 2015 Longevity GWAS Using the *Drosophila* Genetic Reference Panel. Journals Gerontol. Ser. A Biol. Sci. Med. Sci. 70: 1470–1478. 10.1093/gerona/glv047 PMC4631106 25922346

[65] Longdon B., Hadfield J. D., Day J. P., Smith S. C. L., McGonigle J. E. et al, 2015 The causes and consequences of changes in virulence following pathogen host shifts. PLoS Pathog. 11: e1004728 doi: 10.1371/journal.ppat.1004728 25774803 10.1371/journal.ppat.1004728 PMC4361674

[66] Agnello V., Abel G., Elfahal M., Knight G. B., and Zhang Q. X., 1999 Hepatitis C virus and other flaviviridae viruses enter cells via low density lipoprotein receptor. Proc. Natl. Acad. Sci. U. S. A. 96: 12766–71. 10535997 10.1073/pnas.96.22.12766 PMC23090

[67] Albecka A., Belouzard S., de Beeck A. O., Descamps V., Goueslain L. et al, 2012 Role of low-density lipoprotein receptor in the hepatitis C virus life cycle. Hepatology 55: 998–1007. doi: 10.1002/hep.25501 22121002 10.1002/hep.25501

[68] Finkelshtein D., Werman A., Novick D., Barak S., and Rubinstein M., 2013 LDL receptor and its family members serve as the cellular receptors for vesicular stomatitis virus. Proc. Natl. Acad. Sci. U. S. A. 110: 7306–11. doi: 10.1073/pnas.1214441110 23589850 10.1073/pnas.1214441110 PMC3645523

[69] Deng H., Fung G., Shi J., Xu S., Wang C. et al, 2015 Enhanced enteroviral infectivity via viral protease-mediated cleavage of Grb2-associated binder 1. FASEB J. 29: 4523–31. doi: 10.1096/fj.15-274829 26183772 10.1096/fj.15-274829 PMC4608907

[70] Deng H., Fung G., Qiu Y., Wang C., Zhang J. et al, 2017 Cleavage of Grb2-Associated Binding Protein 2 by Viral Proteinase 2A during Coxsackievirus Infection. Front. Cell. Infect. Microbiol. 7: 85 doi: 10.3389/fcimb.2017.00085 28361043 10.3389/fcimb.2017.00085 PMC5352685

[71] Xu J., Hopkins K., Sabin L., Yasunaga A., Subramanian H. et al, 2013 ERK signaling couples nutrient status to antiviral defense in the insect gut. Proc. Natl. Acad. Sci. 110: 15025–15030. doi: 10.1073/pnas.1303193110 23980175 10.1073/pnas.1303193110 PMC3773808

[72] Liu X., Hodgson J. J., and Buchon N., 2017 Drosophila as a model for homeostatic, antibacterial, and antiviral mechanisms in the gut (K. A. Kline, Ed.). PLOS Pathog. 13: e1006277 doi: 10.1371/journal.ppat.1006277 28472194 10.1371/journal.ppat.1006277 PMC5417715

[73] Leibfried A., Fricke R., Morgan M. J., Bogdan S., and Bellaiche Y., 2008 Drosophila Cip4 and WASp define a branch of the Cdc42-Par6-aPKC pathway regulating E-cadherin endocytosis. Curr. Biol. 18: 1639–48. doi: 10.1016/j.cub.2008.09.063 18976911 10.1016/j.cub.2008.09.063

[74] Fricke R., Gohl C., Dharmalingam E., Grevelhörster A., Zahedi B. et al, 2009 Drosophila Cip4/Toca-1 Integrates Membrane Trafficking and Actin Dynamics through WASP and SCAR/WAVE. Curr. Biol. 19: 1429–1437. doi: 10.1016/j.cub.2009.07.058 19716703 10.1016/j.cub.2009.07.058

[75] Valanne S., Wang J.-H., and Ramet M., 2011 The Drosophila Toll Signaling Pathway. J. Immunol. 186: 649–656. doi: 10.4049/jimmunol.1002302 21209287 10.4049/jimmunol.1002302

[76] Chuang H.-C., Lan J.-L., Chen D.-Y., Yang C.-Y., Chen Y.-M. et al, 2011 The kinase GLK controls autoimmunity and NF-κB signaling by activating the kinase PKC-θ in T cells. Nat. Immunol. 12: 1113–1118. doi: 10.1038/ni.2121 21983831 10.1038/ni.2121

[77] Loegering D. J., and Lennartz M. R., 2011 Protein kinase C and toll-like receptor signaling. Enzyme Res. 2011: 537821 doi: 10.4061/2011/537821 21876792 10.4061/2011/537821 PMC3162977

[78] Karlas A., Berre S., Couderc T., Varjak M., Braun P. et al, 2016 A human genome-wide loss-of-function screen identifies effective chikungunya antiviral drugs. Nat. Commun. 7: 11320 doi: 10.1038/ncomms11320 27177310 10.1038/ncomms11320 PMC4865845

[79] Varjak M., Saul S., Arike L., Lulla A., Peil L. et al, 2013 Magnetic fractionation and proteomic dissection of cellular organelles occupied by the late replication complexes of Semliki Forest virus. J. Virol. 87: 10295–312. doi: 10.1128/JVI.01105-13 23864636 10.1128/JVI.01105-13 PMC3754020

[80] Shelly S., Lukinova N., Bambina S., Berman A., and Cherry S., 2009 Autophagy is an essential component of Drosophila immunity against vesicular stomatitis virus. Immunity 30: 588–98. doi: 10.1016/j.immuni.2009.02.009 19362021 10.1016/j.immuni.2009.02.009 PMC2754303

[81] Lamiable O., and Imler J.-L., 2014 Induced antiviral innate immunity in Drosophila. Curr. Opin. Microbiol. 20: 62–68. doi: 10.1016/j.mib.2014.05.006 24907422 10.1016/j.mib.2014.05.006 PMC4133299

[82] Long G., Pan X., Kormelink R., and Vlak J. M., 2006 Functional entry of baculovirus into insect and mammalian cells is dependent on clathrin-mediated endocytosis. J. Virol. 80: 8830–3. doi: 10.1128/JVI.00880-06 16912330 10.1128/JVI.00880-06 PMC1563848

[83] Kataoka C., Kaname Y., Taguwa S., Abe T., Fukuhara T. et al, 2012 Baculovirus GP64-mediated entry into mammalian cells. J. Virol. 86: 2610–20. doi: 10.1128/JVI.06704-11 22190715 10.1128/JVI.06704-11 PMC3302255

[84] Blissard G. W., and Rohrmann G. F., 1990 Baculovirus Diversity and Molecular Biology. Annu. Rev. Entomol. 35: 127–155. doi: 10.1146/annurev.en.35.010190.001015 2154158 10.1146/annurev.en.35.010190.001015

[85] Zhu F., Ding H., and Zhu B., 2013 Transcriptional profiling of Drosophila S2 cells in early response to Drosophila C virus. Virol. J. 10: 210 doi: 10.1186/1743-422X-10-210 23803447 10.1186/1743-422X-10-210 PMC3704779

[86] Merkling S. H., Bronkhorst A. W., Kramer J. M., Overheul G. J., Schenck A. et al, 2015 The epigenetic regulator G9a mediates tolerance to RNA virus infection in Drosophila. (P. F. Vale, Ed.). PLoS Pathog. 11: e1004692 doi: 10.1371/journal.ppat.1004692 25880195 10.1371/journal.ppat.1004692 PMC4399909

[87] Guan X., Middlebrooks B. W., Alexander S., and Wasserman S. A., 2006 Mutation of TweedleD, a member of an unconventional cuticle protein family, alters body shape in Drosophila. Proc. Natl. Acad. Sci. U. S. A. 103: 16794–9. doi: 10.1073/pnas.0607616103 17075064 10.1073/pnas.0607616103 PMC1636534

[88] Graveley B. R., Brooks A. N., Carlson J. W., Duff M. O., Landolin J. M. et al, 2011 The developmental transcriptome of Drosophila melanogaster. Nature 471: 473–479. doi: 10.1038/nature09715 21179090 10.1038/nature09715 PMC3075879

[89] Deddouche S., Matt N., Budd A., Mueller S., Kemp C. et al, 2008 The DExD/H-box helicase Dicer-2 mediates the induction of antiviral activity in drosophila. Nat. Immunol. 9: 1425–1432. doi: 10.1038/ni.1664 18953338 10.1038/ni.1664

[90] Chen Q., Sun L., and Chen Z. J., 2016 Regulation and function of the cGAS–STING pathway of cytosolic DNA sensing. Nat. Immunol. 17: 1142–1149. doi: 10.1038/ni.3558 27648547 10.1038/ni.3558

