# Supplementary figures and images for: Isolation of a natural DNA virus of Drosophila melanogaster, and characterisation of host resistance and immune responses
Source: PLoS Pathog. 2018 Jun 4;14(6):e1007050. doi: 10.1371/journal.ppat.1007050 (PMC6002114; doi:10.1371/journal.ppat.1007050)

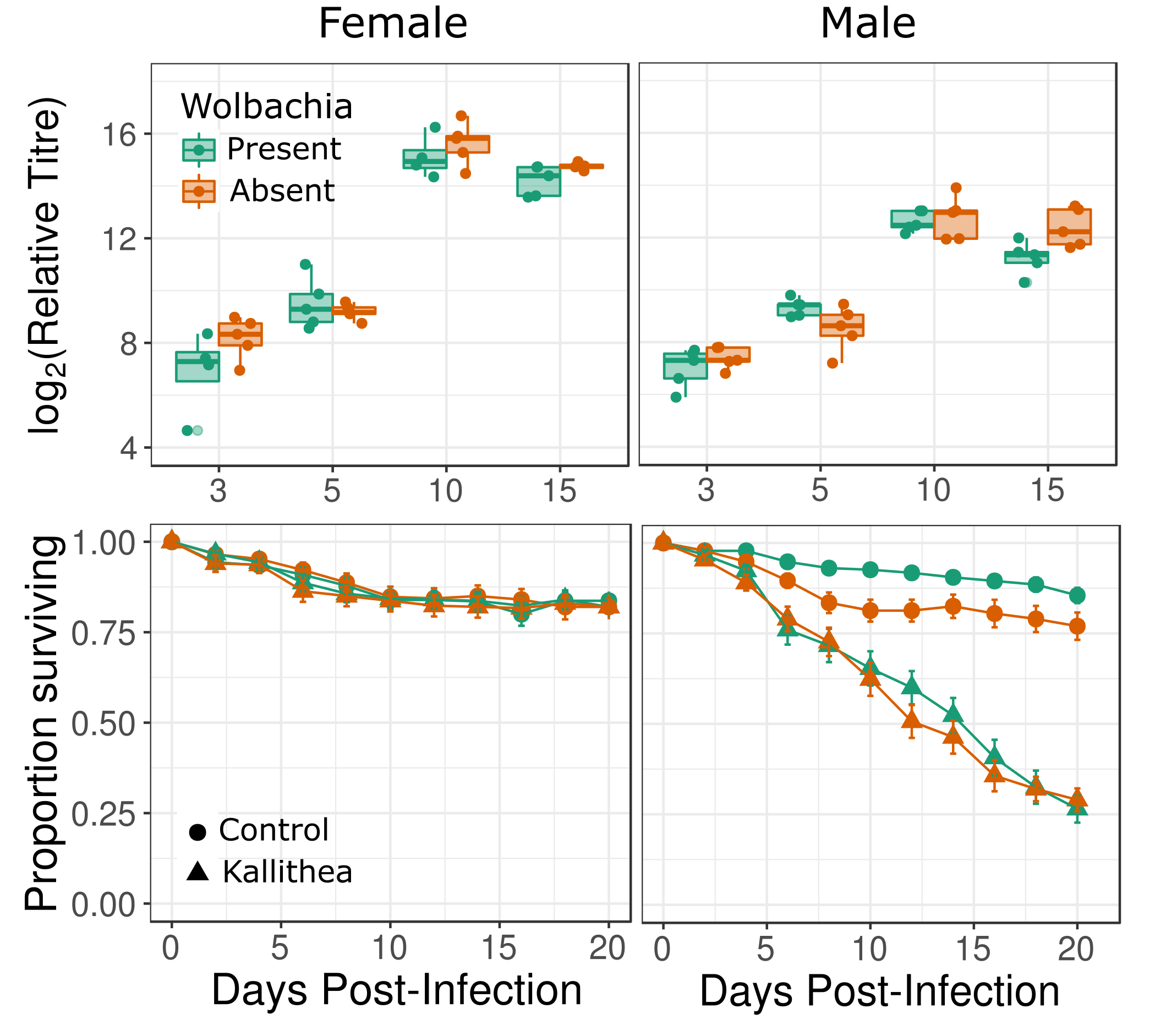

Supplement: S1 Fig — Upper panels: Log-transformed relative viral titre in Wolbachia positive (green) or negative (orange) OreR female and male flies. Lower panels: mortality curves for gradient control-injected (circle) or KV-injected (triangle) OreR female and male flies, with or without Wolbachia. (TIF) [file ppat.1007050.s001.tif]

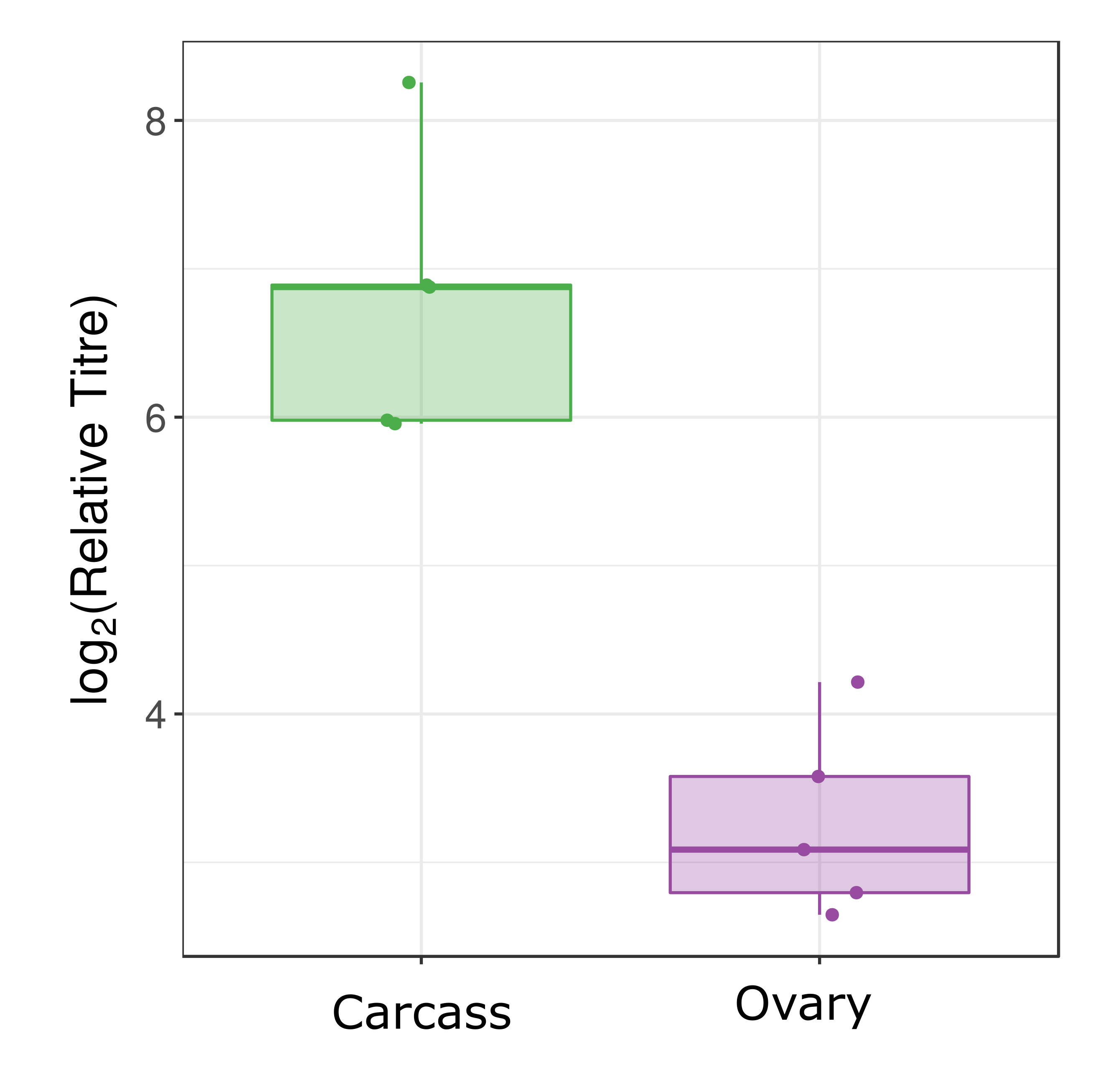

Supplement: S2 Fig — Females had higher viral titres in non-ovary tissues at 3 DPI. However, this could be affected by the route of infection, and the average ploidy of each sample. (TIF) [file ppat.1007050.s002.tif]

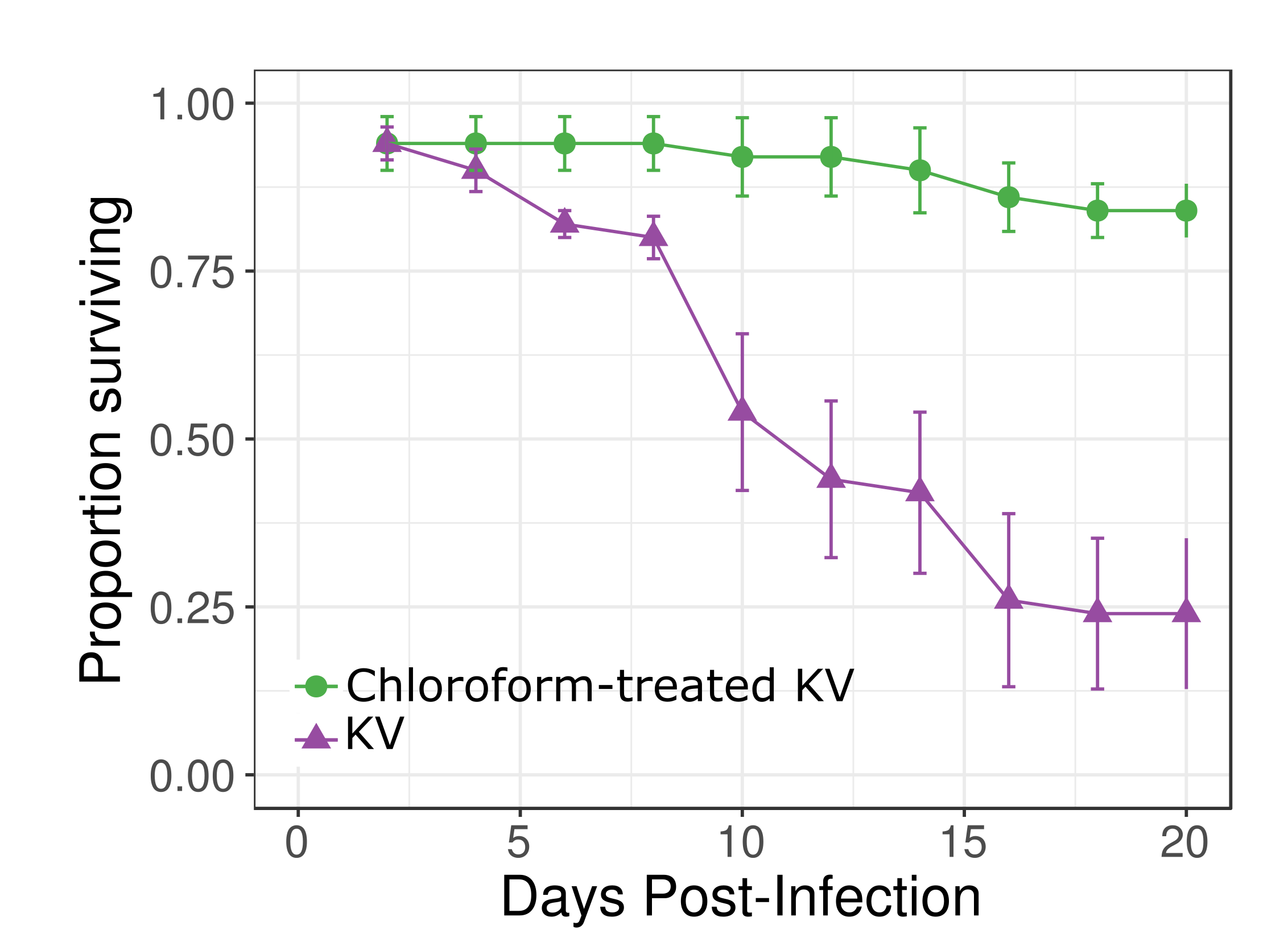

Supplement: S3 Fig — Chloroform-treatment is expected to inactivate enveloped viruses such as KV, but unenveloped viruses (including most +ssRNA viruses) are expected to retain infectivity. We confirmed mortality following KV infection was not caused by contaminating DAV by comparing injection of the KV isolate with (green) or without (purple) inactivating chloroform treatment. (TIF) [file ppat.1007050.s003.tif]

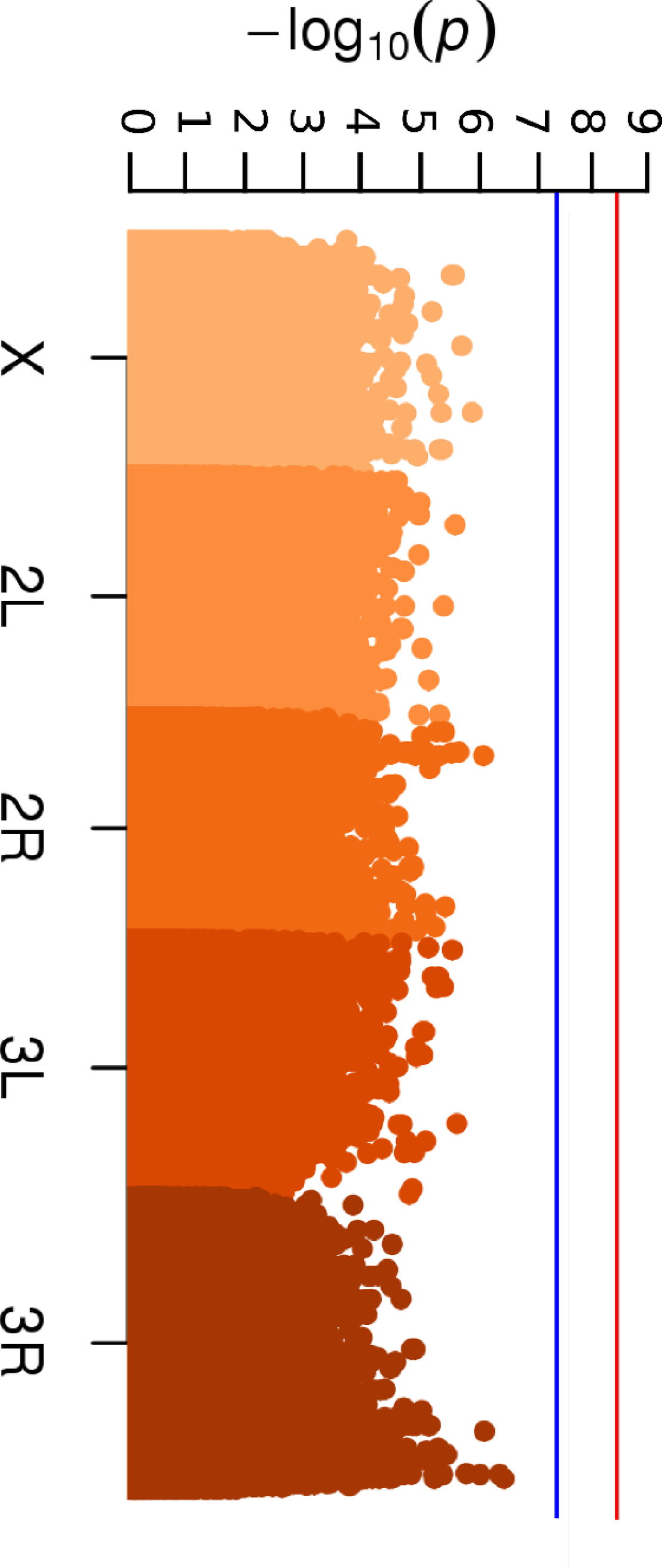

Supplement: S4 Fig — No polymorphism had a significant effect on sex-specific mortality. The blue line denotes prand = 0.05 and the red line is prand = 0.01. (TIF) [file ppat.1007050.s004.tif]

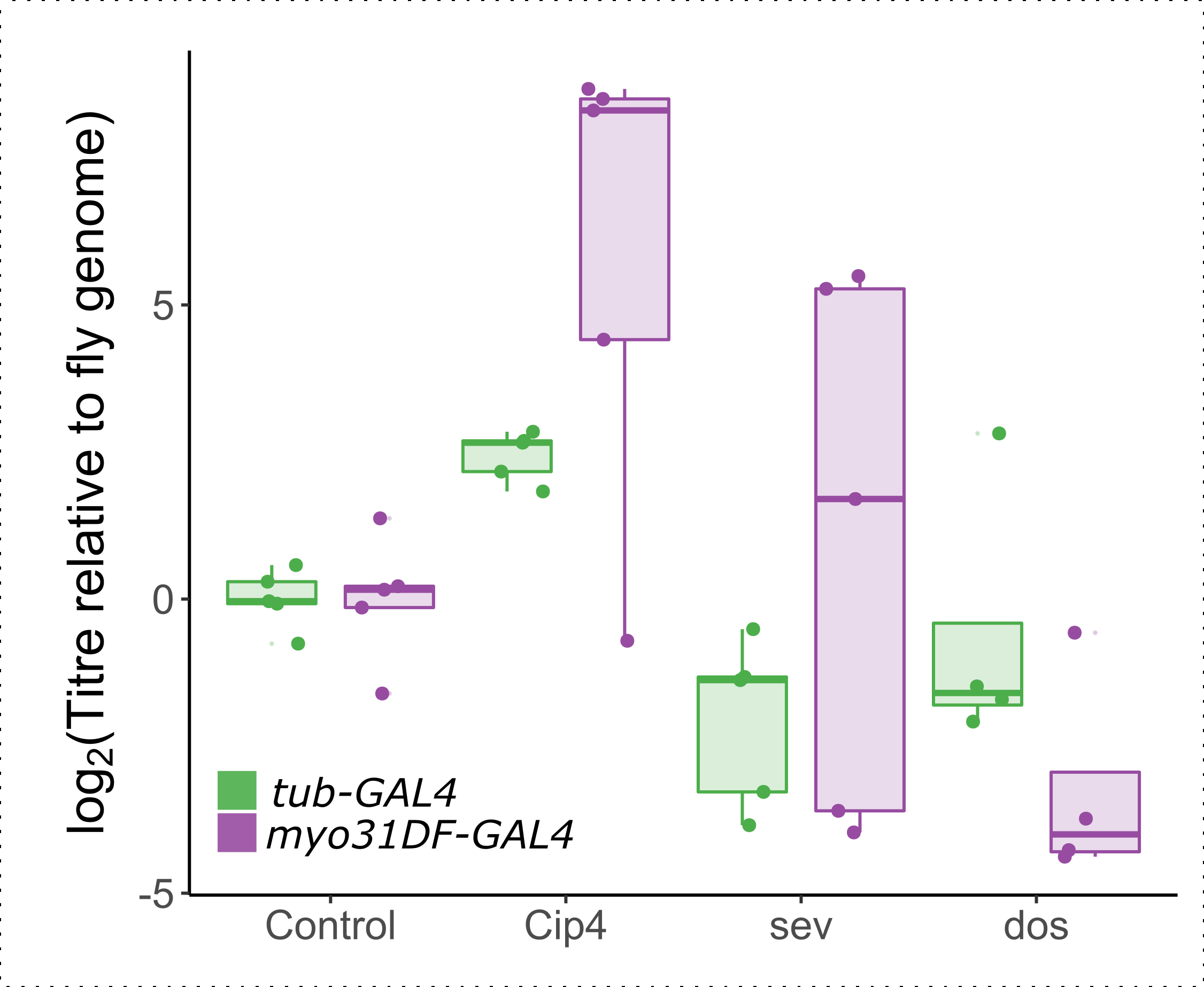

Supplement: S5 Fig — KV titre was measured in flies expressing a foldback hairpin targeting 18 genes identified in the GWAS, using GAL4 lines that knock each down in either the whole fly (tub-GAL4, green) or specifically in the gut (myo31DF-GAL4, purple). Only those causing a significant increase in titre relative to other knock-down lines (e.g. Fig 5) are shown here. Note that the cross between myo31DF-GAL4 and CG12821IR was inexplicably lethal, and that titre was highly variable in some of the other myo31DF-GAL4 crosses. (TIF) [file ppat.1007050.s005.tif]

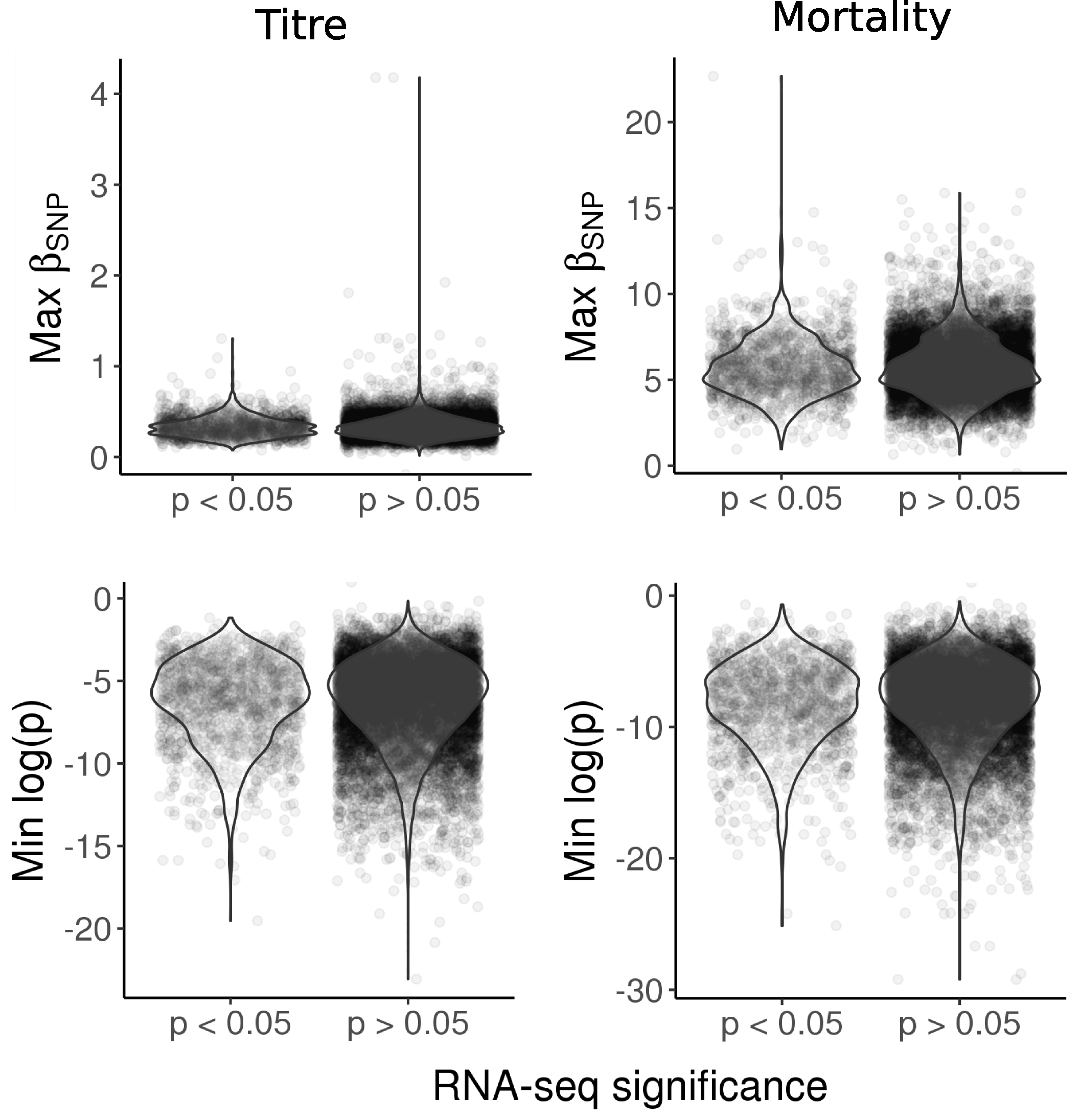

Supplement: S6 Fig — Genes were split into KV-responsive and KV-unresponsive genes based on the RNA sequencing differential expression analysis (p < 0.05). The largest effect size (max βSNP) and lowest p-value was recorded for each gene in each GWAS, and compared between the KV-responsive and unresponsive genes. We find no significant difference between any comparisons. (TIF) [file ppat.1007050.s006.tif]

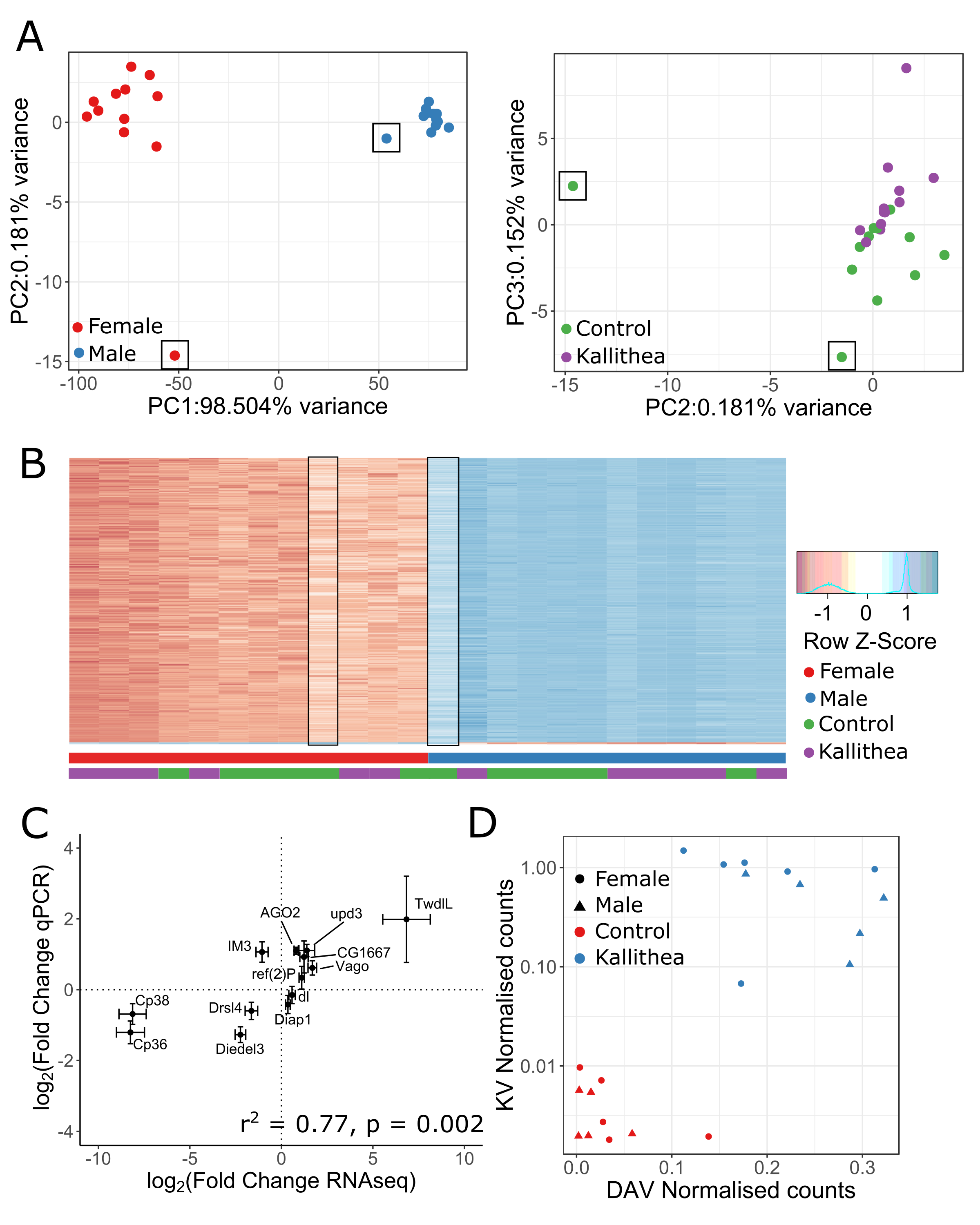

Supplement: S7 Fig — (A) The first three principal components of read counts per gene in RNA-sequencing data, plotted such that each library is represented by a point. Males (blue) and females (red) are separated on PC1. Control-injected (green) and KV-injected (purple) are separated on PC3. For (B), we clustered libraries based on expression of the 1000 most variable genes, where each row on the heatmap is a gene, and the columns are libraries. Together, these analyses identified two possible outlier libraries, which were excluded (black rectangles in A and B). (C) We selected 13 well-studied immune genes and genes with a clear phenotype association (e.g. chorion proteins), distributed across the range of differential expression values, for qPCR verification. Using 5 independent biological replicates from the outbred DGRP population, we confirmed that differential expression for these genes was highly correlated between qPCR and RNA-seq (r2 = 0.77, p = 0.002). (D) We found low-level DAV contamination in our RNA-sequencing experiment. The plot shows the relationship between DAV viral titre and average KV gene expression, where each point is the number of reads mapping to KV and DAV for each library, normalised by library size factor and genome length. Note that KV is plotted on a log10 scale, but DAV on a linear scale. (TIF) [file ppat.1007050.s007.tif]

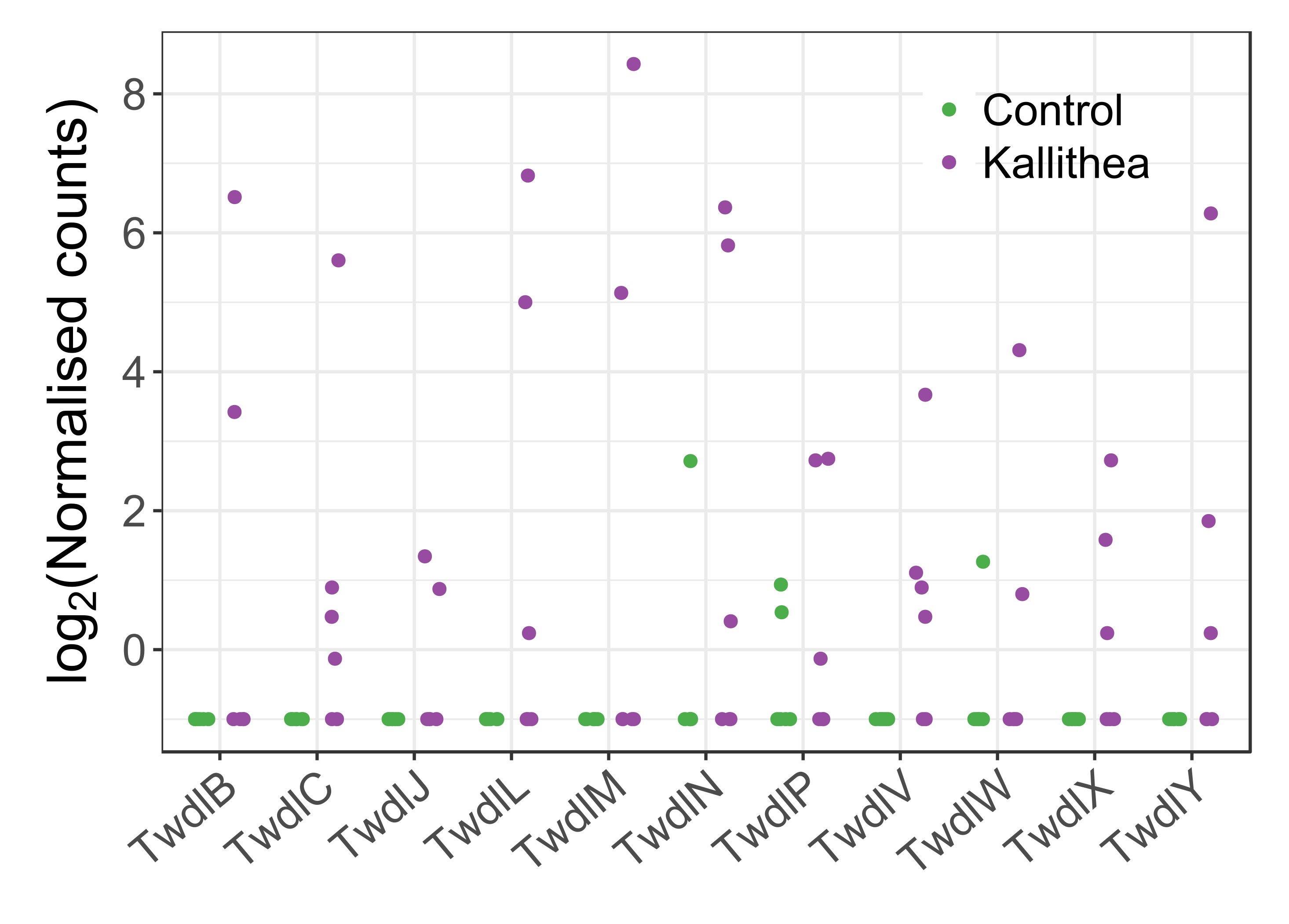

Supplement: S8 Fig — Network analysis identified a pathway marginally enriched for genes identified in these studies (p = 0.03). Shown are interactions (edges) between genes identified in GWAS or RNA-sequencing studies (square nodes), allowing a single gene not included in the original gene list to mediate an interaction (triangular nodes). Genes (nodes) are coloured by functional annotation and white nodes are unannotated. Node labels are associated with the bottom-right corners of nodes. (TIF) [file ppat.1007050.s008.tif]

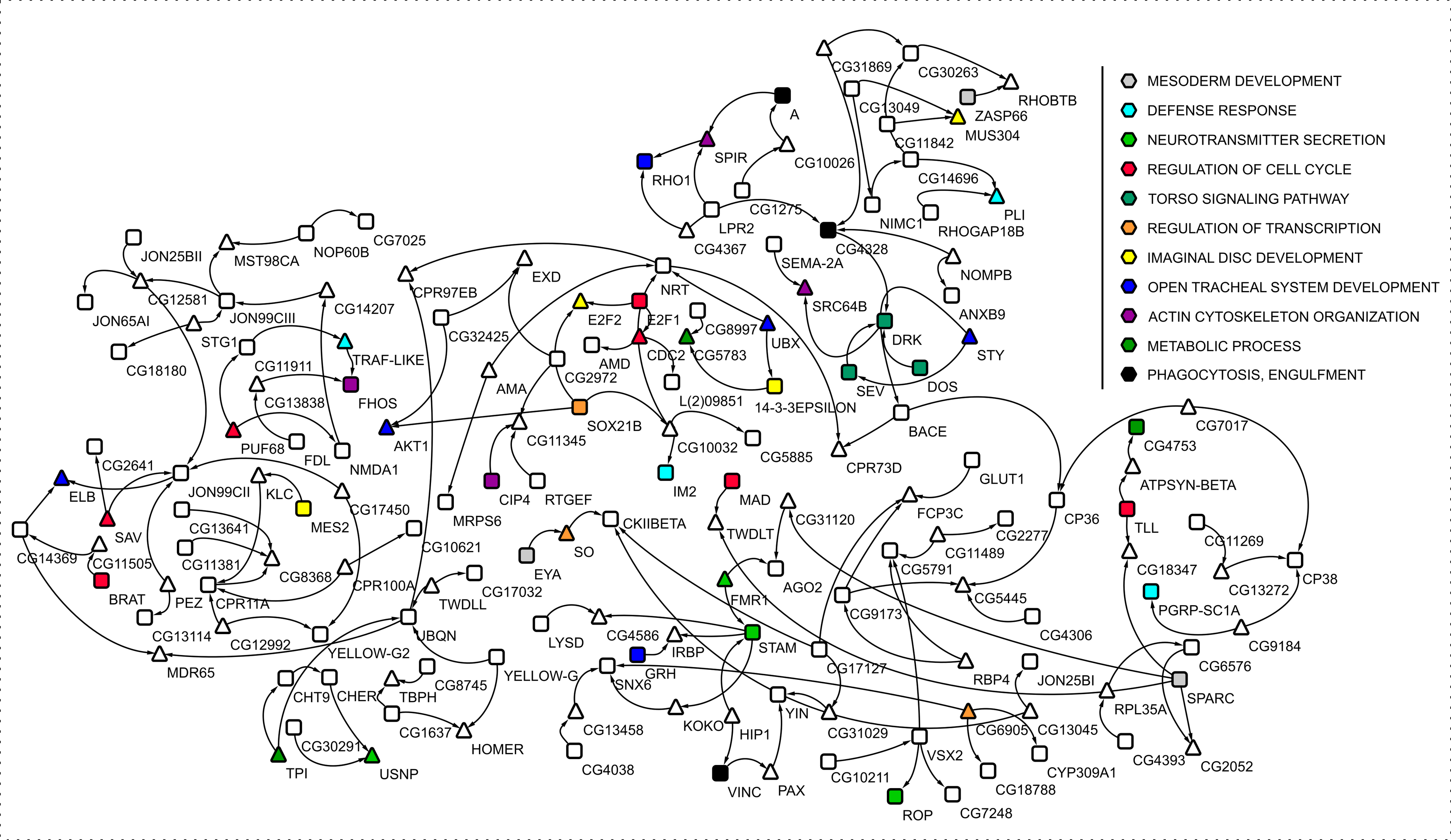

Supplement: S9 Fig — A subset of KV-infected (purple) vials showed very high expression of Tweedle genes, whereas these were mostly unexpressed in control (green) adult flies. (TIF) [file ppat.1007050.s009.tif]

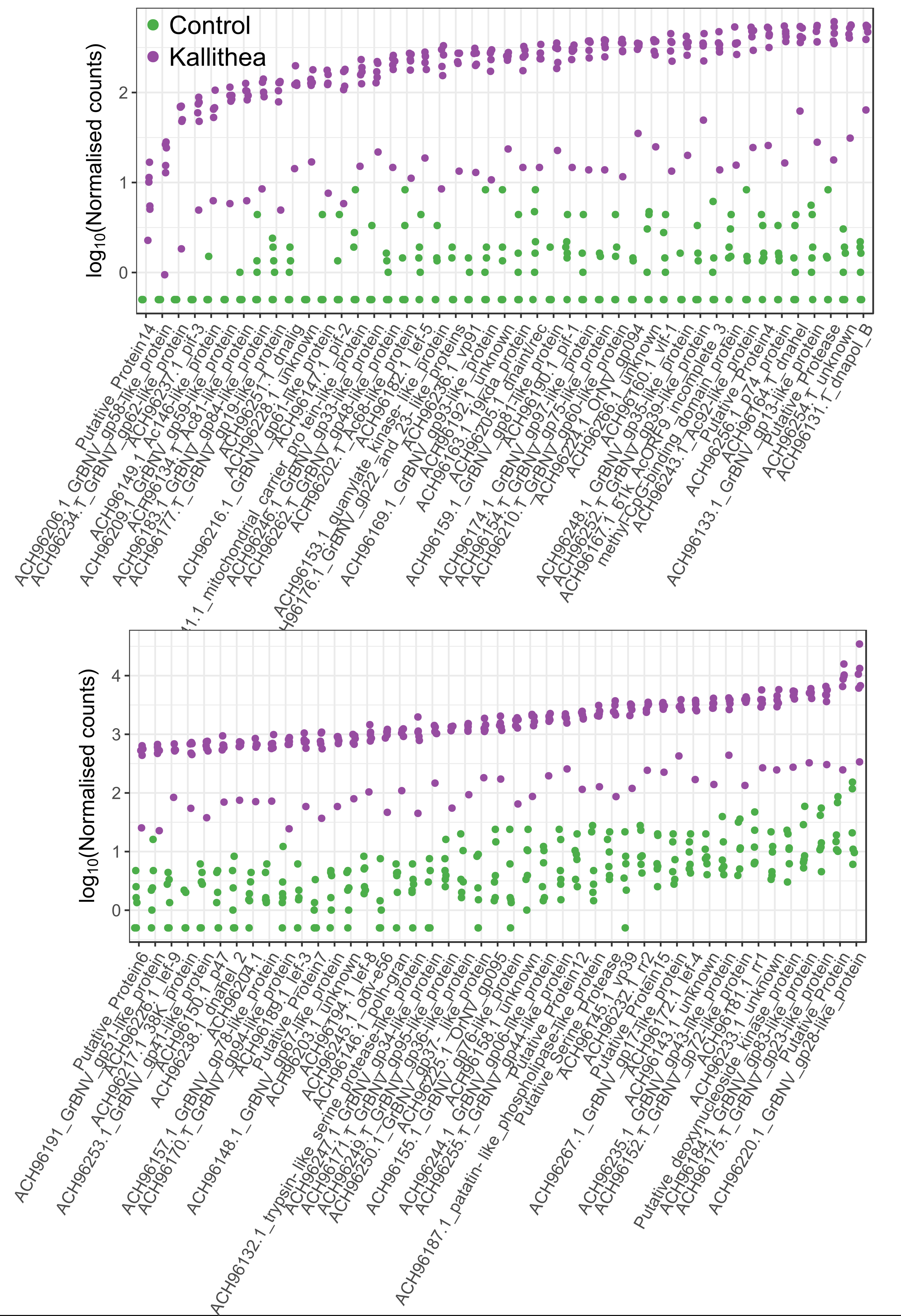

Supplement: S10 Fig — Most KV genes are expressed at 3 DPI. One KV-injected vial of flies had a lower level of infection. Control libraries also showed mapping to KV genes, most likely due to a low level (<0.5%) of barcode switching among libraries run together. The lower panel is a continuation of the upper panel. (TIF) [file ppat.1007050.s010.tif]
